# Optimized multi-epitope neoantigen human cytomegalovirus vaccine based on adenovirus vectors elicits potent antiviral immunity

**DOI:** 10.3389/fimmu.2025.1658220

**Published:** 2025-11-21

**Authors:** Shasha Jiang, Xianjuan Zhang, Heping Zhao, Fengjun Liu, Zonghui Li, Xiaoli Yang, Wenxuan Liu, Jing Lv, Yi Zhang, Yiran Zhang, Yan Yu, Bin Wang, Yunyang Wang

**Affiliations:** 1 Department of Clinical Laboratory, Honghui Hospital, Xi’an Jiaotong University, Xi’an, China; 2 Department of Pathogenic Biology, School of Basic Medicine, Qingdao University, Qingdao, China; 3 Department of Clinical Laboratory, The Affiliated Hospital of Qingdao University, Qingdao, China; 4 Qingdao Municipal Hospital, Qingdao, China; 5 Department of Clinical Laboratory, Chengdu Aerotropolis Asia Heart Hospital, Chengdu, China; 6 Chongqing Hospital, Union Hospital, Tongji Medical College, Huazhong University of Science and Technology, Chongqing, China; 7 Department of Endocrinology and Metabolism, the Affiliated Hospital of Qingdao University, Qingdao, China

**Keywords:** human cytomegalovirus, adenovirus vaccine, immune response, memory T cell, mitochondrial biogenesis

## Abstract

**Introduction:**

Human cytomegalovirus (HCMV) induces severe morbidity and mortality in neonates, organ transplant recipients, and immunocompromised individuals. Currently, there is no licensed vaccine for HCMV. Given its ability to elicit a robust and enduring CD8 T cell response, we designed a recombinant adenovirus vaccine, referred to as the rAdMev vaccine, using bioinformatics methods based on human cytomegalovirus multi-antigen epitopes.

**Methods:**

Five proteins of HCMV (pp150, pp65, gB, gH, IE1) were analyzed using bioinformatics tools, and 58 T cell epitopes, 66 Th cell epitopes, and 15 B cell epitopes were screened out. The immunogenicity of the overlapping candidate peptides was initially tested *in vitro* using molecular docking techniques and flow cytometry. Subsequently, the selected dominant antigenic epitopes were recombined into the adenovirus vector. To enhance the vaccine’s efficacy, we employed a mixed priming schedule, combining an adenoviral vaccine with a protein vaccine. Finally, the immune response induced by heterologous vaccination was analyzed by proteomics.

**Results:**

The developed vaccine demonstrates favorable characteristics in terms of major histocompatibility complex affinity, immunogenicity, and population coverage. Primary immunization of mice with the rAdMev vaccine induces a potent innate immune response, characterized by highly activated dendritic cell subsets and the polarization of macrophages towards the M1 phenotype. Heterologous vaccination fosters the generation of robust polyfunctional (IFN-γ, TNF, IL-2, Granzyme B, Perforin) CD8 T cell responses, leading to the establishment of persistent effector memory T cells. Furthermore, we observed that heterologous vaccination activates fatty acid β-oxidation through the PPAR signaling pathway, enhancing mitochondrial biogenesis and promoting CD8 T cell memory formation *in vivo*.

**Conclusions:**

We have developed a novel multi-epitope recombinant adenovirus vaccine that can elicit long-lasting antiviral cellular immunity, providing new insights into the development of vaccines against HCMV.

## 1 Introduction

Human cytomegalovirus (HCMV), a member of the Betaherpesvirinae subfamily, is ranked as one of the largest human viruses ever identified (1). It establishes a latent infection that typically remains asymptomatic for decades in immune-competent hosts but emerges as the most prevalent and severe disease following conditions, such as human immunodeficiency virus (HIV) infection, solid organ transplantation (SOT), or hematopoietic stem cell transplantation (SCT) (2–4). Moreover, congenital HCMV infection can cause severe and enduring neurological symptoms in newborns (5). While currently available HCMV antiviral drugs, including ganciclovir, cidofovir, and fomivirsen, demonstrate some effectiveness in slowing viral progression, they face criticism for poor bioavailability, significant toxicity and drug resistance after prolonged use (6). Consequently, there is a pressing need for a durable approach to suppress HCMV reactivation and its sequelae. Several HCMV vaccine candidates that have entered clinical trials mainly include subunit vaccines, DNA or RNA-based vaccines, and whole-virus vaccines (including live-attenuated, attenuated, or disabled infectious single-cycle viruses) (7). Among them, ASP0113, a DNA vaccine developed by Astellas, consists of two plasmids encoding gB and ppUL83. However, in a Phase II study involving organ transplant recipients, the vaccine failed to demonstrate efficacy against CMV disease, and its development has been discontinued (8). Another two-component alphavirus replicon particle vaccine, which expresses HCMV gB or a pp65/IE1 fusion protein, was able to elicit antibody and T-cell responses in a Phase I trial in HCMV-seronegative individuals, yet it has not been advanced further (9). In addition, a whole-virus chimeric vaccine constructed by genomic recombination between the Towne strain and the clinical isolate Toledo strain showed no evidence of enhanced immune responses in a Phase I study involving seropositive subjects (10). The pursuit of a vaccine against HCMV has been ongoing for more than 50 years. Unfortunately, no licensed vaccines are currently available (11).

There is growing evidence emphasizing the significance of inducing polyfunctional T-cell immunity for effectively controlling persistent viral infections (12). Notably, HCMV triggers a massive T-cell response that persist in the human body, migrate to peripheral tissues, and maintains its effector function. Key contributors to this response are CD4 T cells, capable of modulating primary HCMV infection, restricting ongoing replication within specific tissues, and stimulating antibody responses (13). The generation of HCMV-specific CD4 T cells is intimately correlated with disease protection in patients. While CD8 T cells offer superior protection to immunocompromised individuals by effectively curbing viral reactivation, CD8 T cell responses to specific HCMV antigens deviate from conventional kinetics after the resolution of acute HCMV infection (14). This deviation, termed memory inflation, involves the contraction of the majority of antigen-specific CD8 T cells, forming a persistent central memory pool with a multifunctional effector memory phenotype (15).

Intriguingly, memory T cells, including those specific to HCMV antigens, exhibit the ability to generate faster and more robust responses upon encountering antigens. Metabolically, memory CD8 T cells exhibit a significant spare respiratory capacity (SRC) (16, 17). It is noteworthy that CD8 memory T cells exhibit a higher mitochondrial mass compared to their naive counterparts. Upon activation, these memory T cells show an augmented capacity for fatty acid oxidation (FAO) and oxidative phosphorylation (OXPHOS), sustaining this increase to a greater extent than immature T cells (18). This suggests that the increased mitochondrial mass in memory T cells not only enhances their oxidative capacity but also their glycolytic capacity, and the greater mitochondrial mass confers a bioenergetic advantage to memory T cells, facilitating their rapid recall response upon reinfection (19). Hence, promoting mitochondrial biogenesis and enhancing energy metabolism via vaccination represents a crucial strategy for establishing enduring immune memory.

Viral vectors represent promising tools for gene therapy and vaccines (20). Vaccines based on viral vectors can enhance immunogenicity without adjuvants and induce potent cytotoxic T lymphocyte (CTL) responses to eliminate virus-infected cells (21). Human Adenovirus serotype 5 (Ad5) as a gene delivery vector has been extensively studied due to its ability to be easily produced at high titers (22). Recombinant adenovirus vector vaccines have been validated in clinical trials targeting HIV-1, influenza, COVID-19 and solid tumors (23–26). Given the high immunogenicity, safety, and efficient amplification of adenovirus vector vaccines, we have selected adenovirus as the vector for the development of an HCMV vaccine. However, a well-known challenge in the development of adenovirus vector vaccines, particularly for Ad5, is the prevalence of pre-existing immunity in the population, which hinders their effectiveness (27). To address the hurdle of pre-existing anti-Ad5 antibodies, heterologous prime-boost regimens and prolonged prime-boost intervals have been frequently used to enhance the immune response (28).

HCMV IE1 protein is silenced during the latent state and essential for HCMV reactivation (29). Furthermore, it serves as an immunodominant target for specific CTL, playing a crucial role in controlling viral replication and reactivation from latency (30). Additionally, the envelope protein pp65, a primary component of mature viral particles, shares the ability to trigger cellular immune responses, contributing to the regulation of HCMV replication, similar to the IE1 protein (31). Phosphoprotein 150, the second most abundant envelope protein after pp65, exhibits high immunogenicity and plays a crucial role in the assembly and release of viral particles (31). It is also one of the primary proteins that elicit cellular immune responses.

Recent studies have revealed the essential roles of glycoprotein B (gB) and glycoprotein H (gH) in cell fusion and viral entry, making them prominent targets for neutralizing antibodies, essential for preventing initial infection (32). However, deficient CD8 T cell responses may potentially limit gB and gH in stimulating antibody production.

In this study, bioinformatics methods were employed to identify highly homologous peptides in both mice and humans. These tandem peptides were then homologously recombined into adenovirus vectors to prepare a novel HCMV vaccine. Heterologous boosting of the rAdMev vaccine resulted in a robust polyfunctional T-cell response. This response included cytotoxicity to infected cells, T cell expansion and differentiation, and the establishment of virus-specific memory T cells. Furthermore, we discovered mitochondrial proteome remodeling inducing energy metabolism in memory T cells. This metabolic adaption contributes to the induction of a more potent, broader, and enduring immune response. Our research findings establish a strong preclinical foundation for developing HCMV vaccines.

## 2 Methods

### 2.1 Prediction of T cell and B cell Epitopes

**Figure 1:**
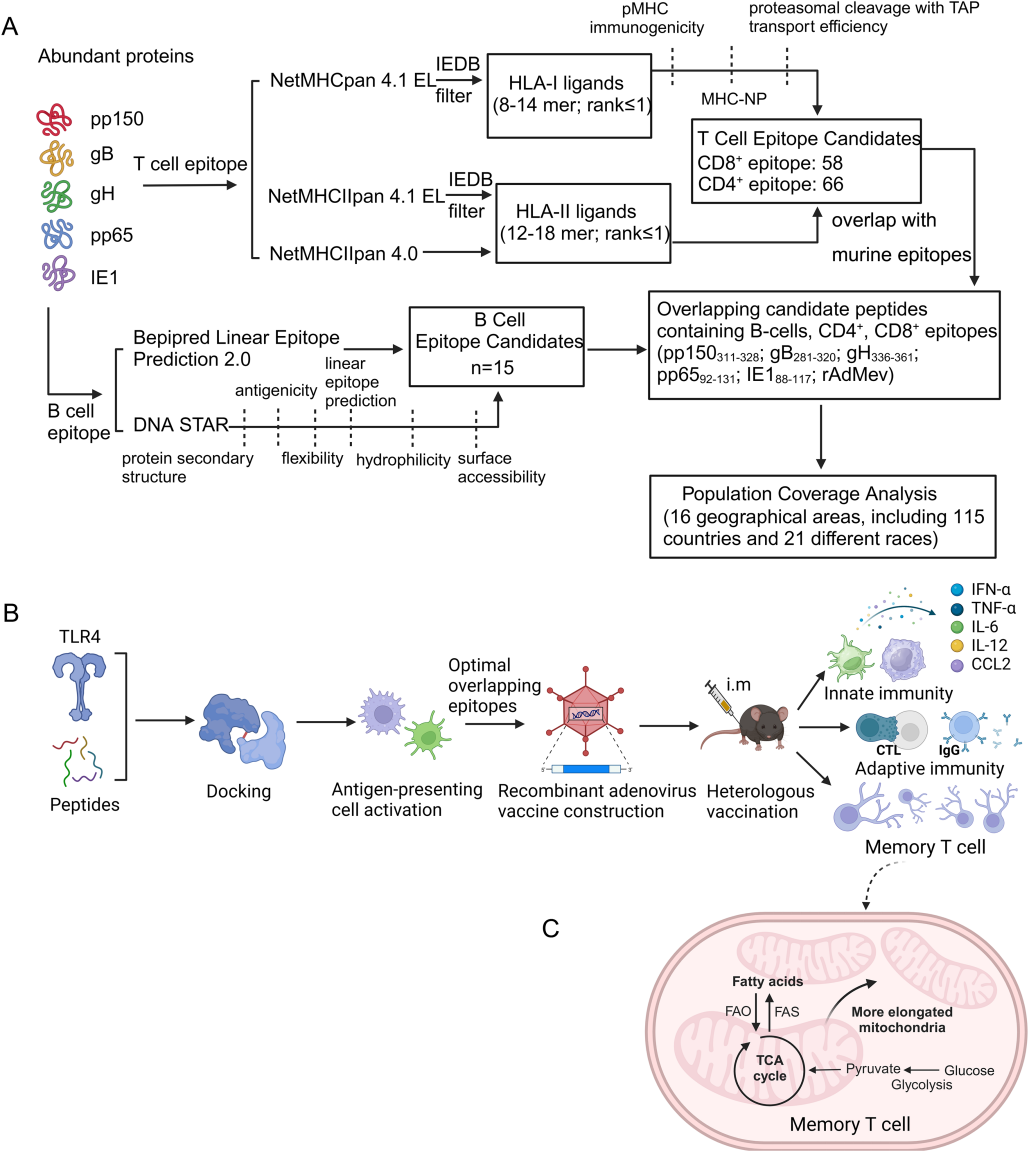
Visual summary of T and B cell epitope prediction and validation of multiple epitope-based recombinant adenovirus vaccines. **(A)** Overview of CD4/CD8 T cell and B cell epitope prediction workflows. We employ bioinformatics to analyze five proteins derived from human cytomegalovirus to examine the coverage of high-frequency HLA alleles and the immunogenicity of the predicted epitopes. B-cell epitope regions were followed by filtering for protein secondary structure, antigenicity, flexibility, linear epitope prediction, hydrophilicity and surface accessibility. **(B)** The screened overlapping epitope peptides were recombined into adenovirus vectors, as well as mice were inoculated heterologously to assess the safety and effectiveness of the vaccine. **(C)** Metabolic traits of memory T cells. FAO and OXPHOS maintain the survival and energy requirements of T cells during memory T cell differentiation.

T cell epitope prediction was performed employing the five proteins for the reference HCMV AD169. Protein accession identification numbers are as follows: pp150: ACL51112.1, gB: ACL51135.1, gH: ACL51144.1, pp65: ACL51152.1, IE1: ACL51183.1. HLA class I binding predictions were performed using the NetMHCpan EL 4.1 available at the Immune Epitope Database and Analysis Resource (IEDB-AR, http://tools.iedb.org/) and selected the 11 HLA class I alleles with the highest global population proportion (**Supplementary Table S1**), combined the peptide prediction of pMHC I immunogenicity and proteasomal cleavage with TAP transport efficiency (33). We selected epitopes with percentile rank ≤ 1 and the alignment length ranged from 8 to 14 mers (34). The probability of peptide being naturally processed and combined to MHC molecule was evaluated with the MHC-NP tool (http://tools.iedb.org/mhcnp/), and finally the screened T cell epitope peptides corresponding to multiple alleles were reduced to a single occurrence, and the overlapping short peptides were accommodated within longer sequences, up to 14 mers in length (**Figure 1**, **Supplementary Table S1**).

For CD4 T cell epitopes, IEDB MHCII Binding and NetMHCIIpan4.0 (http://www.cbs.dtu.dk/services/NetMHCIIpan-4.0/), were used for prediction with a predicted length of 12–18 mers. We settled on a 1% rank value for peptides, a threshold related to the top 1% score obtained from random natural peptides (35), and used this threshold to identify 66 candidate peptides **Figure 1** and **Supplementary Table S2**).

The B cell epitope predictions were carried out using the Bepipred 2.0 algorithm (cut off of ≥0.5) (36) provided with the IEDB (**Figure 1**, **Supplementary Table S3**). The hydrophilic epitopes on the protein surface are preferred. DNAstar (https://www.dnastar.com/software/) further identifies B cell epitopes, including protein secondary structure, antigenicity, flexibility, linear epitope prediction, hydrophilicity, and surface accessibility.

We also identified peptides derived from viral proteins predicted to bind murine MHC coded for by H2-D^b^, H2-K^b^, and H2-IA^b^ haplotypes (37). For the prediction of cell epitopes for murine MHC I/II genotypes, we employed the NetMHCpan EL 4.0 algorithm available in the IEDB database to align sequences of HCMV gB, pp150, pp65, gH, and IE1, with alignment length ranged from 8 to 14mers. To minimize the chance of epitope omission, we further utilized the SYFPEITHI database (https://syfpeithi.de/60-PredictEpitope.htm; http://g6altair.sci.hokudai.ac.jp/g6/service/pocasa/) for prediction, with alignment length ranged from 8 to 14 mers. We then selected longer sequence regions that were recognized by humans and mice to be used as vaccine antigens. The dominant epitope sequences for mice are provided in **Supplementary Table S4**.

### 2.2 Population coverage analysis

The distribution of HLA alleles varies in different countries around the world and consequently affects the valid response to vaccines. We employed a population coverage analysis tool embedded in IEDB (http://tools.iedb.org/population/) to predict the population coverage of MHC class I, MHC class II, and class combined for each epitope in 16 geographical areas, including 115 countries and 21 different races.

### 2.3 Construction of rAdMev

The screened overlapping epitopes were concatenated with the GGSGGGSGS linker and then homologous recombination was performed into the E1 region of the adenovirus type 5 genome, meanwhile the E3 region of the genome was deleted to obtain a non-replicating recombinant human adenovirus type 5 vaccine based on multiple Epitope. The GGSGGGSGS linker mitigates the connection to other protein regions and enhances stability through efficient segregation (**Table 1**).

**Table 1:** HCMV candidate peptides List.

HCMV antigen	Sequence
pp150_311-328_	GSAFSSVPKKHVPTQPLD
gB_280-320_	NGTNRNASYFGENADKFFIFPNYTIVSDFGRPNAAPETHR
gH_336-360_	RRTVEMAFAYALALFAAARQEEAGTE
pp65_92-131_	VNVHNPTGRSICPSQEPMSIYVYALPLKMLNIPSINVHHY
IE1_88-117_	QIKVRVDMVRHRIKEHMLKKYTQTEEKFTG
Mev	GSAFSSVPKKHVPTQPLDGGSGGGSGSNGTNRNASYFGENADKFFIFPNYTIVSDFGRPNAAPETHRGGSGGGSGSRRTVEMAFAYALALFAAARQEEAGTEGGSGGGSGSVNVHNPTGRSICPSQEPMSIYVYALPLKMLNIPSINVHHYGGSGGGSGSQIKVRVDMVRHRIKEHMLKKYTQTEEKFTG

GGSGGGSGS used as a linker.

### 2.4 Molecular docking with TLR4 receptors

The 3D structures of dominant epitope peptides and tandem proteins were predicted utilization the ColabFold (https://colab.research.google.com/) homologous modeling method (38). ColabFold provides accelerated prediction of protein structure and complexes by associating the rapid homology search of MMseqs2 with AlphaFold2 or RoseTTAFold (38). The polypeptide structure was drawn using ChemBioDraw Ultra 14.0 and subsequently imported into ChemBio3D Ultra 14.0 for energy minimization, with the Minimum RMS Gradient set to 0.001. POCASA 1.1 (http://g6altair.sci.hokudai.ac.jp/g6/service/pocasa/) was employed for the prediction of protein binding sites, while AutoDock Vina 1.1.2 (https://vina.scripps.edu/downloads/) and HDock (http://hdock.phys.hust.edu.cn/) were utilized for the docking of TLR4 (PDB ID: 4G8A) with the polypeptide and Mev, respectively. In parallel, the docked complexes predicted models were visualized by pymol.

To further assess the potential of HCMV candidate polypeptides to activate TLR4 receptors, the peptides were incubated with RAW264.7 and DC2.4 cell lines for 48 hours. Subsequently, total cellular proteins were extracted from the cells, and Western blotting was performed to evaluate the activation of surface TLR4 receptors on antigen-presenting cells. The antibody information is as follows: TLR4 (Santa Cruz, cat. sc-293072) and β-Tubulin (Abclonal, cat. AC021).

### 2.5 Vaccine toxicity evaluation

It is imperative to develop vaccines that possess high antigenicity, non-allergenicity, and non-toxicity. In order to evaluate the safety of these vaccines, the body weight and food intake of heterologous and homologous vaccinated mice were monitored for one week after the second immunization. Simultaneously, mouse serum was collected for biochemical analysis to assess potential toxicity on vital organs such as the heart (serum creatine kinase and lactate dehydrogenase), liver (ALT, AST), and kidney (UA). All samples were subjected to three independent experiments utilizing a fully automated biochemical analyzer.

### 2.6 Mice and immunization

Female C57BL/6 mice from 6 to 8 weeks were bred in the animal facility at Qingdao University in accordance with Institutional Animal Care and Use Committee guidelines (approval number: N0.20220928C5716820230223119) and randomly assigned to different vaccine groups, which included PBS group, heterologous vaccination group (AP) and the homologous vaccination group (AA). The multiple epitope-based human adenovirus serotype 5 vaccine (rAdMev) and multi-antigen epitope vaccine (Mev) were synthesized by Sangon Biotech (Shanghai, China) and GenScript Biotechnology Co., Ltd. (Hong Kong, China), respectively.

For immunization, mice (n=6 in each group) were injected intramuscularly. Immunize with one dose of rAdMev (1×10^8^ PFU) at day 0, followed by rAdMev (1×10^8^ PFU) or Mev (20 µg protein with AddaVax^™^ adjuvant in a volume ratio of 1:1) at days 60, respectively. AddaVax^™^ was purchased from InvivoGen (Toulouse, France), which is a squalene-based oil-in-water nano-emulsion with a formulation similar to that of MF59^®^. All mice in this study were maintained under specific pathogen-free conditions with 12 light/12 dark cycles, at a temperature of approximately 18-23°C, and a humidity level maintained between 40-60%.

### 2.7 Cell culture

DC2.4 cell line and RAW264.7 cell line (ATCC) were cultured in high glucose Dulbecco’s Modified Eagle’s medium (DMEM, Gibco) containing 10% fetal bovine serum (FBS, Gibco), 1% penicillin and streptomycin, and maintained at 37°C with 5% CO_2_.

### 2.8 *In vitro* dendritic cell and macrophage activation assessment

*In vitro*, RAW264.7 cells and DC2.4 cells were cultured according to the above method and were seeded in 96-well plates at a density of 5 × 10^4^ cells per well and stimulated with pp150_311-328_, gB_280-320_, pp65_92-131_, gH_336-360_, IE1_88–117_ and Mev proteins (5 μg/ml) for 48 hours. The expression of cell surface markers CD80, CD86, CD40, MHCI, and MHCII was detected by flow cytometry. The antibodies used were as follows: Brilliant Violet 650-CD80 (Biolegend, cat. 104731), Pacific Blue-CD86 (Biolegend, cat. 105021), APC-CD40 (Biolegend, cat. 124611), PerCP/Cyanine5.5-H-2Kb (Biolegend, cat. 116515), and Brilliant Violet 605- I-A/I-E (Biolegend, cat. 107639). Fluorescence measurements were taken on a Beckman CytoFLEX instrument and analyzed using Flow Jo V10.5.3.

### 2.9 Analysis of the innate immune response

Mice were immunized with rAdMev, and draining inguinal lymph node from mice was harvested on days 1, 3, and 7 after immunization and digested with 1 mg/ml type IV collagenase (Absin, abs47048004) for 20 minutes at 37°C, followed by filtration through 100 μm filters to obtain a single-cell suspension. Single-cell samples were stained using Pacific Blue-CD3 (Biolegend, cat. 100213), FITC-CD11c (Biolegend, cat. 117305), Brilliant Violet 605-MHCII (Biolegend, cat. 107639), APC-CD11b (Biolegend, cat. 101211), Brilliant Violet 605-CD103 (Biolegend, cat. 121433), APC/Cyanine7-CD8a (Biolegend, cat. 155015), Brilliant Violet 650-CD86 (Biolegend, cat. 105035), PE/Cyanine7-F4/80 (Biolegend, cat. 123113), PerCP/Cyanine5.5-CD206 (Biolegend, cat. 141715), and PE anti-Nos2 (iNOS, Biolegend), cat. 696805 antibodies. Fluorescence data was acquired on a Beckman CytoFLEX instrument and analyzed using Flow Jo V10.5.3. On the first day following the initial immunization, mouse serum was isolated. Elisa kit was used to detect the expression of cytokines including CCL2 (R&D Systems, cat. MJE00B), IL-12p70 (Abcam, cat. ab119531), IL-6 (Abcam, cat. ab222503), IFN-α (Thermo Fisher Scientific, cat. BMS6027), and TNF-α (Abcam, cat. ab100747).

### 2.10 Evaluation of antigen-specific T cell response

Fourteen days after the second immunization, mice were euthanized by cervical dislocation. Spleens and draining lymph nodes were isolated to prepare single-cell suspensions (n=6). Subsequently, cell surface staining was performed using APC/Cyanine7-CD8a (Biolegend, cat. 155015), PE/Cyanine7-CD69 (Biolegend, cat. 104511), and APC-CD107a (LAMP-1, Biolegend, cat. 121614) antibodies to assess the activation status of CD8 T cells. On the other hand, 5 × 10^4^ splenocytes per well were seeded into 96-well plates, followed by a 12-hours incubation with Mev protein (5 μg/mL), in the presence of brefeldin A (Biolegend, cat. 420601) at 37°C. The percentage of cytokine production by effector T cells was assessed by surface and intracellular staining using a Fixation/Permeabilization Solution Kit (Thermo, cat. GAS004). The antibody cocktail comprises the following fluorescently labeled antibodies: Pacific Blue-CD3 (Biolegend, cat. 100213), APC/Cyanine7-CD8a (Biolegend, cat. 155015), Brilliant Violet 605-CD4 (Biolegend, cat. 116027), PerCP/Cyanine5.5-IFN-γ (Biolegend, cat. 505821), PE/Cyanine7-TNF-α (Biolegend, cat. 506323), APC-Granzyme B (Biolegend, cat. 372203), PE-Perforin (Biolegend, cat. 154305), FITC-IL-2 (Biolegend, cat. 503805), PE-IL-4 (Biolegend, cat. 504103) and APC-IL-6 (Biolegend, cat. 504507).

### 2.11 T cell proliferation assessment

Cell growth was assessed via the Cell Counting Kit-8 (CCK8) assay. Briefly, 14 days following the second immunization, mouse spleen T lymphocytes were seeded in 96-well plates at 2 × 10^4^ cells per well, and the cells were treated with 5 ug/mL Mev protein for 48 and 72 hours. Following incubation, 10 μl of CCK8 (Abcam, ab228554) solution was added to each well and incubated at 37°C for 2 hours, and then absorbance was measured at 450 nm using a SPECTROstar Nano microplate reader (BMG Labtech).

### 2.12 Specific antibody assay

Detection of specific antibodies generated by homologous and heterologous vaccination by ELISA. Biotinylated protein (Biotin-Mev) was prepared by Sangon Biotech (Shanghai, China). Elisa plates were precoated at 4°C with 0.5 μg of streptavidin in 50 mM NaHCO_3_ (pH 9.6) for 12 hours, followed by blocking with 5% bovine serum albumin (BSA) for 2 hours at 37°C. Subsequently, biotin-Mev was incubated with streptavidin for 1 hour at 37°C. Serum from immunized mice was collected every 7 days via suborbital venous, diluted with 5% BSA, and incubated with Biotin-Mev at 37°C for 1 hour according to different serum dilution ratios. HRP-labeled IgG (1:2000, Abcam), IgG1 (1:2000, ABclonal), and IgG2a (1:2000, ABclonal) antibodies were incubated for 1 hour at 37°C. Following incubation, 100 μl of TMB substrate solution (Sigma, catalog number T0440) was added. After 15 minutes, 1M H_3_PO_4_ was introduced to terminate the reaction, and the optical density (OD) was measured at 450 nm. The highest sample dilution corresponding to an OD value of the experimental group/negative control group ≥ 2.1 was determined.

### 2.13 Neutralization

Serum from day 74 mice were incubated at 56 °C for 30 minutes to inactivate the complement. Subsequently, the serum was diluted proportionally, mixed with equal volumes of AD169 strain (MOI = 1), and incubated at 37°C for 1 hour. The virus mixture that had undergone treatment was incubated with MRC-5 cells at 37°C, 5% CO_2_. After 48 hours, cells were fixed with 4% paraformaldehyde for 10 minutes, permeabilized with 0.5% Triton X-100 for 5 minutes, and blocked with 1% BSA for 1 hour at room temperature. The cells were incubated with HCMV IE1/2 antibody overnight, followed by a subsequent incubation with a secondary antibody (Thermo Fisher Scientific, catalog A10521) for 1 hour in the dark and the nuclei were stained with 1 μg/mL 4’,6-diamidino-2-phenylindole (DAPI) for 5 minutes. Neutralizing titers (ID50) were determined using the Reed and Muench method (39).

### 2.14 Memory T cell activation analysis

Fourteen days after the second immunization, mouse blood was collected via the infraorbital vein to isolate peripheral blood mononuclear cells (PBMC). Subsequently, mice were euthanized by cervical dislocation, and spleens and draining lymph nodes were isolated to prepare single-cell suspensions (n=6). The subtypes and activation status of memory T cells was employed to assess by Flow cytometry, and utilizing the following fluorescently labeled antibodies: Pacific Blue-CD3 (Biolegend, cat. 100213), APC/Cyanine7-CD8a (Biolegend, cat. 155015), FITC-CD44 (Biolegend, cat. 156007), PE/Cyanine7-CD62L (Biolegend, cat. 156007), PE/Cyanine7-CD69 (Biolegend, cat. 104511), and FITC-CD103 (Biolegend, cat. 110907).

### 2.15 Proteomic analysis

The proteome analysis was conducted similarly as previously described (39). CD8 T lymphocytes were isolated from the draining lymph nodes of mice subjected to various immunization protocols. Total cellular proteins were extracted, followed by enzymatic digestion (40) and TMT labeling of peptides. Labeled peptides were fractionated and analyzed on an Orbitrap 480 (Thermo) mass spectrometer. The quantitative information of the target protein set was normalized. Subsequently, the Complexheatmap R package (R Version 3.4) was utilized to classify the two dimensions of samples and protein expressions, generating a hierarchical clustering heat map. The sorted heatmap of the log2 fold-changes was generated using SPIN (41). The CELLO method (http://cello.life.nctu.edu.tw/) was employed for predicting subcellular localization. Additionally, Blast2GO was used for annotating the target protein set with GO annotations, and the KAAS (KEGG Automatic Annotation Server) software was utilized to perform GO annotation for the target protein set, including KEGG pathway annotation.

### 2.16 Western blotting

The sorted CD8 T cells were lysed with radioimmunoprecipitation assay (RIPA) buffer, which contained a cocktail of protease inhibitors, phenylmethylsulfonyl fluoride (PMSF), and phosphatase inhibitors. Cell lysates were separated through SDS-PAGE gel electrophoresis and transferred to polyvinylidene fluoride (PVDF) membranes (Millipore, Burlington, MA). The membranes were blocked with 5% BSA for 2 hours. Following this, they were incubated with primary antibodies overnight at 4 °C and with secondary antibodies for 2 hours at room temperature on the next day. Following incubation, an electrochemiluminescence solution was applied for 1 minute, and the signal was detected using a chemiluminescence instrument (Imagequant LAS500, Cytiva, USA). The primary antibodies used included S1c27a1 (Abclonal, cat. A12847), Acsl1 (Abclonal, cat. A22737), Cpt2 (Abclonal, cat. A12426), Acoxl (Abclonal, cat. A21217), and β-actin (Abclonal, cat. AC038). The secondary antibody employed was goat anti-rabbit IgG-HRP (Beyotime, cat. A0208).

### 2.17 Immunofluorescence

Sections of paraffin-embedded draining lymph nodes were subjected to a series of steps including deparaffinization, rehydration, and a 5-minute immersion in water before antigen retrieval was performed in a microwave oven using sodium citrate buffer (pH 6.0) for 10 min. Repaired sections were blocked with a solution consisting of 5% bovine serum albumin and 0.05% Triton X-100 (Thermo, cat. 85111) for 30 minutes, followed by overnight incubation with primary antibodies at 4 °C. The secondary antibody (1:500) was incubated at room temperature in the dark for 1 hour and then washed with PBS containing Tween (PBST). Afterward, the sections were incubated with DAPI containing a fluorescence inhibitor for 5 minutes and then sealed with a coverslip. The primary antibodies were as follows: Tomm20 (Abclonal, cat. A11308), Atp5h (Abcam, cat. ab110275), and Cycs (Abcam, cat. ab110325). The secondary antibodies employed were goat anti-mouse-Cy3 (Beyotime, cat. A0521) and goat anti-rabbit fluorescein isothiocyanate (Abcam, ab6717). Image acquisition was performed using an Olympus Fluoview FV1200 microscope.

### 2.18 Electron microscope

To investigate the morphological and quantitative changes of mitochondria at the ultrastructural level, the sorted T cells were fixed with 2.5% glutaraldehyde, then dehydrated and embedded. Imaging was done using transmission electron microscopy (JEOL), with mitochondria observed in randomly selected fields of view within each sample. The micrographs were analyzed using Volocity 3D image analysis software (PerkinElmer).

### 2.19 Mitochondrial respiratory function assay

We assessed mitochondrial respiratory function by measuring the oxygen consumption rate (OCR). Differences in respiratory capacity under various treatment conditions were examined using a real-time high-resolution respirometer (Oxygraph-2k, Austria), following the protocol previously described (42, 43). OCRs were measured, normalized to protein concentration, and expressed as pmol of O_2_ per second per mg of protein. Data acquisition and analysis were performed with DatLab software. Sort mouse spleen CD8 T lymphocytes 14 days after second immunizations, grind the cells with a grinding pestle and expose the mitochondria to 2.1 ml of mitochondrial respiration medium MiR06, which contains 0.5mM EGTA, 20 mM HEPES, 110 mM sucrose, 10 mM KH_2_PO_4_, 20 mM taurine, 60mM K-lactobionate, 3mM MgCl_2_.6H_2_O, 1 mg/ml BSA, catalase 280 U/ml, pH 7.1.

Substrates and inhibitors were added sequentially to tissue homogenates to assess the function of mitochondrial complexes I and II. The specific steps are as follows: the tricarboxylic acid cycle produces a large amount of NADH, and 2 M glutamic acid (G) + 0.8 M malic acid (M) is added to maintain the proton leak stage of complex I. By adding 0.5 M ADP (D) + 1 M MgCl_2_, ATP synthase is activated, leading to the conversion of ADP into ATP and the initiation of oxidative phosphorylation in mitochondrial complex I. The assessment of mitochondria’s capability to perform normal oxidative phosphorylation was accomplished by the addition of 1 M succinate (S). In addition to measuring basal respiration, ATP production was calculated by subtracting respiratory oxygen consumption at 4 mg/ml oligomycin from basal respiration. Mitochondrial maximum electron transfer capacity (maximum respiratory capacity) was determined by 1mM uncoupling agent U (FCCP). Simultaneously, mitochondrial backup respiratory capacity was calculated by subtracting ATP production from maximum respiratory capacity. The measurement of oxidative phosphorylation in mitochondrial complex II was measured by adding 1 mM rotenone.

### 2.20 Statistical analysis

All statistical analyses were conducted using GraphPad Prism 9.0, and the data were presented as mean ± SEM. Flow cytometry fluorescence data were acquired on a Beckman CytoFLEX instrument and analyzed using Flow Jo V10.5.3. For comparisons between two groups, an unpaired Student’s t-test was employed, while comparisons involving three groups utilized one-way analysis of variance (ANOVA), followed by Tukey-Kramer *post hoc* analysis. Statistical significance was defined as *P* < 0.05.

## 3 Results

### 3.1 Global population coverage analysis

Population coverage analysis was conducted for various geographical areas; focusing on the selected dominant epitopes. This analysis included separate evaluations for class I, class II, and a combination of class I and class II epitopes. The results revealed distinct responses among populations in different regions to the candidate peptides (**Supplementary Figure S1**).

Notably, the combined class I and class II epitopes showed coverage of over 84.40%, in regions such as East Asia, Northeast Asia, South Asia, Southeast Asia, Europe, West Indies, North America, and Oceania (**Supplementary Figures S1A, B**). Among these regions, Europe, North America, and Oceania exhibit the highest average coverage and PC90 (**Supplementary Figure S1C**).

### 3.2 Molecular docking

**Figure 2:**
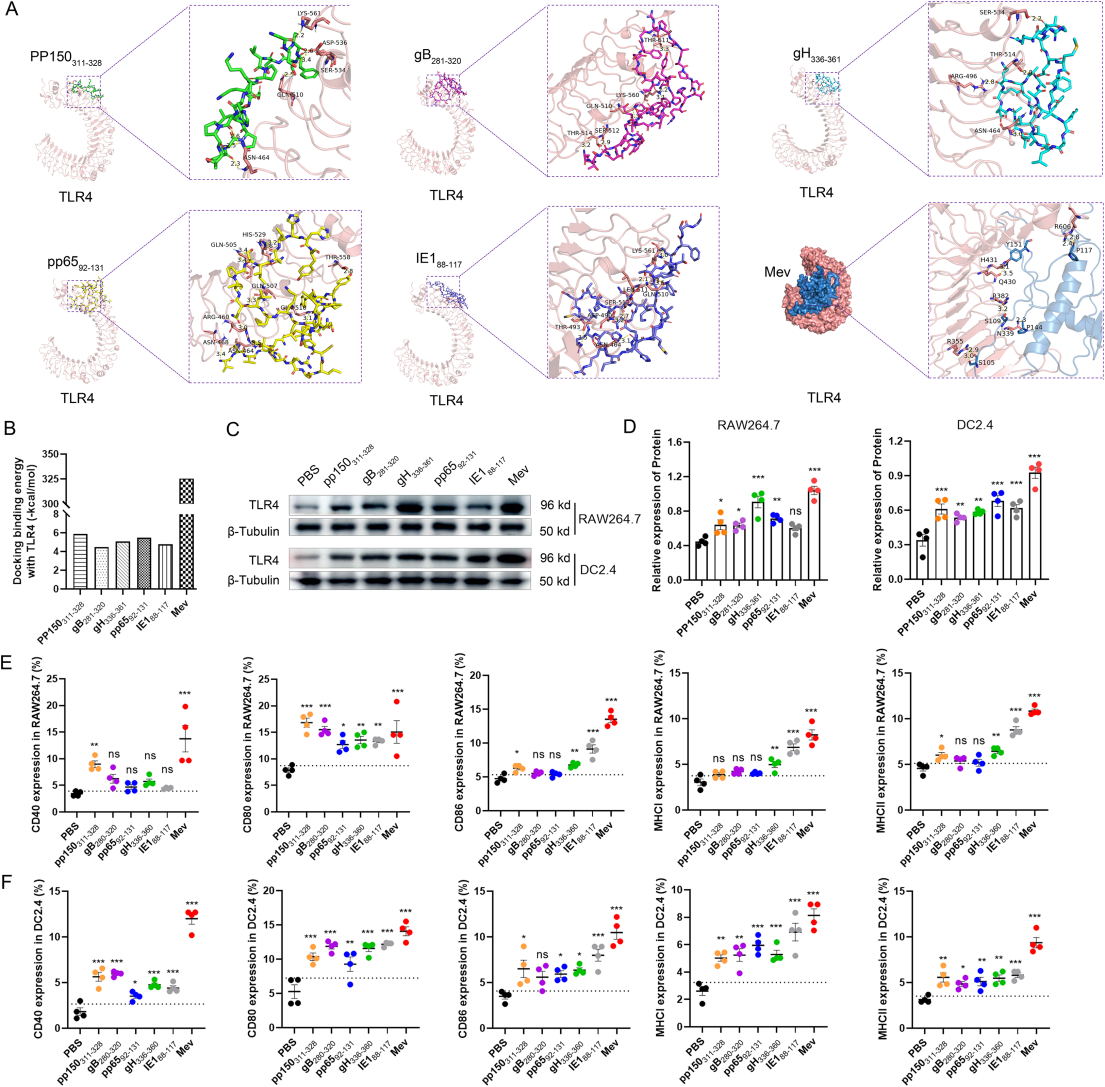
Molecular docking diagrammatic sketch. **(A)** Molecular docking of the candidate peptides pp150_311-328_, gB_280-320_, gH_336-360_, pp65_92-131_, and IE1_88–117_ and Mev individually with TLR4 (PDB ID: 4G8A). The structure of Mev protein is displayed in blue, and the TLR4 receptor structure is displayed in pink. The protein-protein interaction residues are highlighted on the right and the hydrogen bonds are indicated by the yellow dashed lines. **(B)** Evaluation of binding energies released through the molecular docking. After incubation of HCMV candidate peptides with DC2.4 and RAW264.7 cells for 48 hours, the surface expression of TLR4 receptors was detected using Western blotting. β-Tubulin served as an internal reference, while PBS acted as the negative control **(C, D)**. The percentages of surface markers CD80, CD86, CD40, MHCI and MHCII were detected by flow cytometry **(E, F)**. All experimental groups were compared with PBS to analyze statistical differences. The dotted black lines indicate the mean of the negative control group plus 2 standard errors. Each experiment was independently conducted at least three times. One-Way ANOVA followed by Tukey’s test was applied in (D–F)^*^P < 0.05, ^**^P < 0.01, ^***^P < 0.001.

Molecular docking was used to assess the interaction between candidate peptides and the innate immune receptor TLR4, aiming to activate innate immunity and enhance the Th1-type immune response (**Figures 2A, B**). The docking results indicate that the binding of TLR4 to Mev results in the highest energy release, measured at −325.61 kcal/mol, compared to other peptides.

Visualization of the docking complex reveals the deep binding of Mev in the TLR4 center, forming hydrogen bonds with residues around the protein–protein interaction interface, thereby stabilizing the complex (**Figure 2A**). Notably, residues R355, N339, R382, H431, and R606 of TLR4 receptors form hydrogen bonds with S105, P144, S109, Y151, and P117 of Mev, with bond lengths of 3.0 Å, 2.9 Å, 2.3 Å, 3.2 Å, 3.5 Å, 3.1 Å, 2.4 Å, and 2.8 Å, respectively (**Figure 2**).

Furthermore, Western blotting was used to detect the activation of TLR4 receptors on the surface of antigen-presenting cells. Studies have demonstrated that all candidate peptides can activate TLR4 receptors in different degrees, with Mev exhibiting the most significant effect, consistent with the results of computer simulations (**Figures 2C, D**).

### 3.3 Mev activates antigen presenting cells *in vitro*

To assess the ability of multiple antigenic epitope peptides and individual peptides to induce *in vitro* maturation of antigen-presenting cells (APCs), DC2.4 and RAW264.7 cells were stimulated with these peptides. After 48 hours, antigen-presenting cell activation was evaluated by flow cytometry. For T cell activation, APCs need to simultaneously present two crucial signals: the first through a cognate antigen and the second via a costimulatory molecule (44). Our results demonstrated that the expressions of surface markers, including CD80, CD86, CD40, MHCI, and MHCII, were significantly elevated in DC2.4 and RAW264.7 cells treated with Mev compared to the PBS group (**Figures 2E, F**). In contrast, the single peptide group also induced APCs activation, albeit to a lesser extent than the Mev group. According to our *in vitro* data, co-incubation with Mev enhances the phagocytosis of APCs and promotes the upregulation of costimulatory molecules in these cells.

### 3.4 rAdMev induces a robust innate immune response in draining lymph nodes

**Figure 3:**
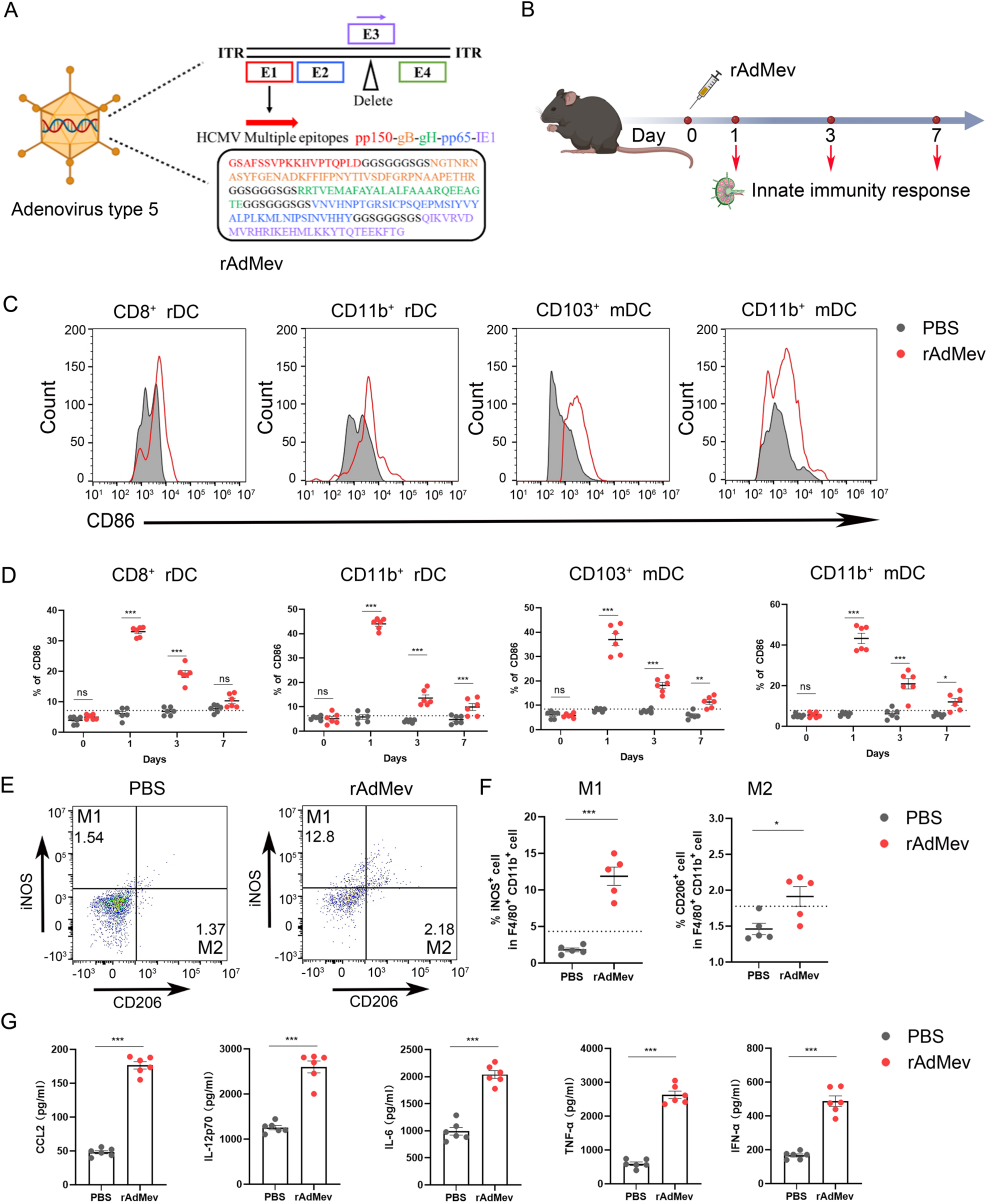
rAdMev construction and activation of innate immune responses. **(A)** Construction of recombinant human adenovirus type 5 vaccine based on multi-epitopes. **(B)** Procedure for the immunization of mice with rAdMev. **(C)** Activation of innate cells at day 1 after rAdMev immunization in dLNs indicated by the upregulation of the activation marker CD86. **(D)** Dynamics profile of innate cell activation at days 1, 3, and 7 after rAdMev immunization. **(E, F)** Assessment of macrophage polarization and activation were executed on the first day post-immunization. **(G)** ELISA was used to profile serum cytokine expression on day 1 following rAdMev immunization. Each experimental group consisted of six mice, and all tests were conducted independently at least three times, with results being presented as the mean ± SEM. The dotted black lines indicate the mean of preimmune response plus 2 standard errors. Statistical analyses were performed using one-way ANOVA, followed by Tukey’s test, and statistical significance was established at ^*^P < 0.05, ^**^P < 0.01 and ^***^P < 0.001.

The rapid initiation of innate immune responses forms the foundation for the development of adaptive vaccine-mediated immunity (45). To elucidate the innate immune responses elicited by rAdMev, mice were immunized on day 0, followed by the detection of the activation status of innate immune cells within the draining lymph nodes (**Figures 3A, B**). After immunization, macrophages and the DC subset, including CD8a^+^ rDC, CD11b^+^ rDC, CD103^+^ mDC, and CD11b^+^ mDC, were highly activated, as evidenced by increased expression of the activation marker CD86 (**Figures 3C, D**). The expression frequencies of DC subtypes increased significantly on day 1 and these innate cell types remained activated until day 3 of immunization, gradually reverting to baseline levels by day 7 (**Figure 3D**). Similarly, within the rAdMev group, there was pronounced activation of macrophages compared to the PBS group. Notably, the proportion of M1 phenotype cells within the rAdMev group significantly exceeded that of the M2 phenotype (**Figures 3E, F**). This observation implies that rAdMev has the capacity to induce a phenotypic shift in macrophages, promoting their polarization toward the M1 phenotype. Subsequently, we analyzed serum cytokine responses on day 1 following immunization using ELISA. Immunization with rAdMev elicited the release of numerous cytokines, including CCL2, IL-12, IL-6, TNF-α, and IFN-α (**Figure 3G**).

### 3.5 Vaccine safety study

As mentioned above, multiple antigenic epitope peptides (Mev) including five overlapping peptides from HCMV pp150_311-328_, gB_281-320_, gH_336-360_, pp65_92-131_, and IE1_88–117_ were selected to construct rAdMev. The experimental groups included the PBS group, the heterologous vaccination group (AP, the first dose of recombinant adenovirus vaccine rAdMev, and the second dose of multiantigenic epitope vaccine Mev), and the homologous vaccination group (AA, where all two doses were recombinant adenovirus vaccine rAdMev). After the second dose of booster immunization, the latent toxicity of the vaccination to the cordis, liver and renal functions of mice was reflected by detection the serum biochemical indicators in mice (**Supplementary Figure S2A**). Among other biochemical parameters, it is similar to the PBS group. Mice weight and percentage of food intake were measured for seven consecutive days after immunization to assess vaccine safety (**Supplementary Figures S2B, C**). As compared with the PBS group, the different vaccination methods showed no significant difference in weight and food intake of mice, revealing a high safety profile for the rAdMev vaccine.

### 3.6 Adaptive immune response induced by rAdMev vaccination

**Figure 4:**
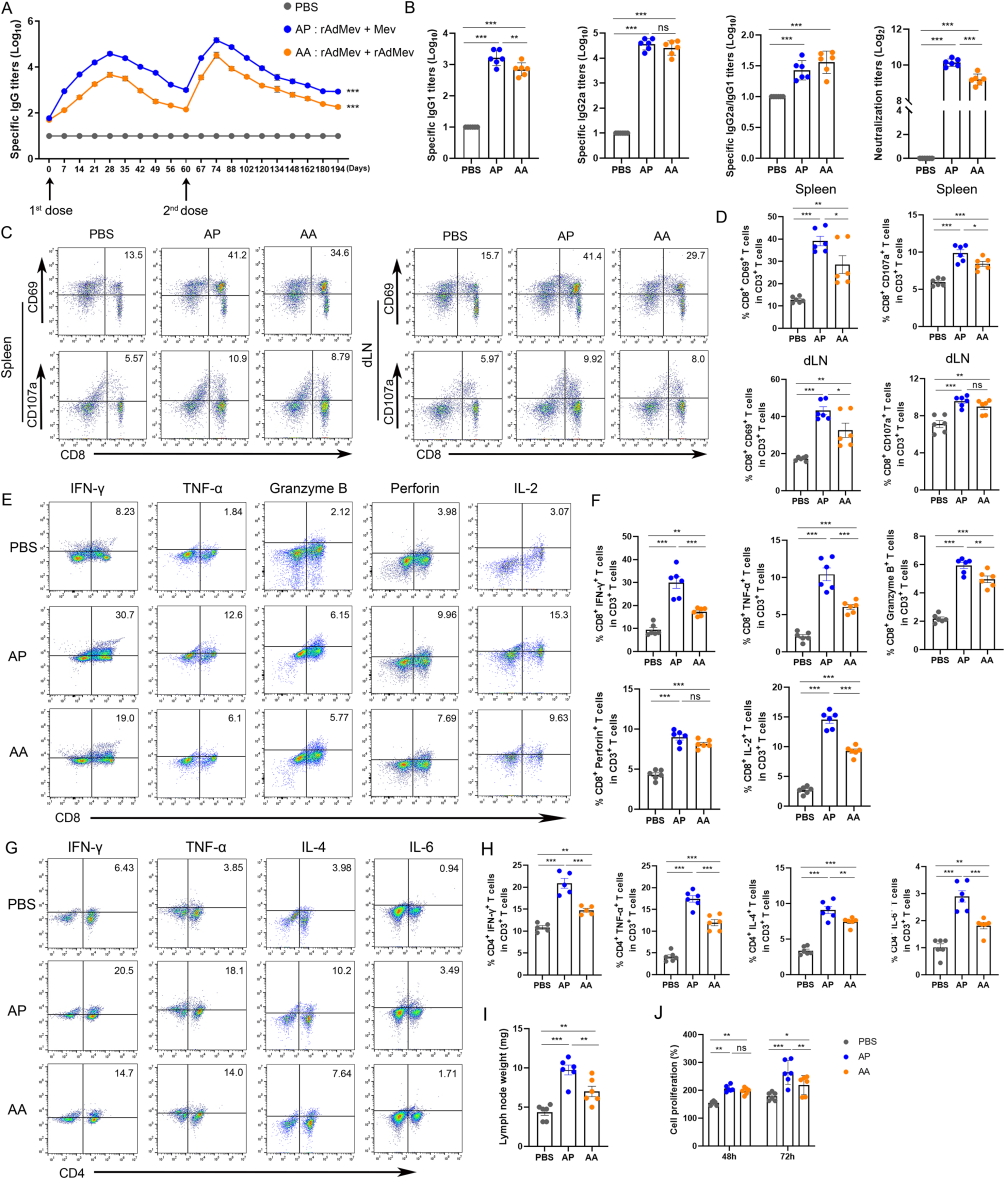
T cell and humoral immunity response induced by rAdMev vaccination in mice. **(A)** Dynamic monitoring of specific IgG antibodies induced following vaccinations. **(B)** On the fourteenth day subsequent to second immunizations, serum samples were collected from mice, and ELISA was employed to assay the production of specific antibodies, including IgG1 and IgG2a, IgG2a to IgG1 ratio, as well as determine the Neutralizing antibodies. **(C, D)** Antigen-specific CD8 T cell responses in draining lymph nodes and spleen at day 14 following rAdMev homologous and heterologous booster immunization. **(E, F)** Antigen-specific CD8 T activation was assessed by detecting the secretion of IFN-γ, TNF-α, granzyme, perforin, and IL-2 by FACS intracellular cytokine staining assay. **(G, H)** The immune response of CD4 T cells was evaluated through the quantification of their secreted cytokines, including IFN-γ, TNF-α, IL-4, and IL-6. **(I)** Draining lymph nodes in the groin of mice were isolated and their weights were measured. **(J)** T-cell proliferation was assessed using CCK8 assay at both 48-hour and 72-hour time points. Each experiment was independently conducted at least three times, n=6. Statistical differences were assessed with One-Way ANOVA followed by Tukey’s test. ^*^P < 0.05, ^**^P < 0.01, ^***^P < 0.001.

C57BL/6 mice underwent an intramuscular immunization with a rAdMe on day 0, followed by either a homologous booster with rAdMev (AA) or a heterologous booster with Mev (AP) at day 60 (**Figure 4**). To further evaluate differences in humoral immunity provoked by heterologous and homologous vaccinations, we continuously monitored the production of specific antibodies in mouse sera. The peak of IgG-specific antibodies was reached 14 days after the second immunization, and induced significantly higher levels of IgG-specific antibodies than the second (**Figure 4A**). Heterologous vaccination produced better antibody levels than homologous vaccination, including neutralizing antibodies, IgG1, and IgG2a (**Figure 4B**). The IgG2a: IgG1 ratio indicated a more Th1-oriented immune response. Collectively, these data suggested that heterologous boosters are more efficient at eliciting multiple specific antibody clones, associated with enhanced antiviral antibody responses. Subsequently, antigen-specific T cell responses were measured in both the spleen and draining lymph nodes 14 days after the administration of the second dose. Our findings revealed detectable antigen-specific CD8 T-cell responses in these tissues, that include CD69 upregulation, degranulation (CD107a) production (**Figures 4C, D**), draining lymph nodes enlargement, and CTL proliferation (**Figures 4I, J**). Significantly, the heterologous inoculation (AP) exhibited superior efficacy compared to its homologous counterpart (AA), consistent with previous investigations. *In vitro* stimulation of T cells with overlapping peptide pools *in vitro*, demonstrated that heterologous vaccination can produce peptide-specific CD8^+^ T cells, secreting an array of immunomodulatory molecules, including IFN-γ, TNF-α, granzyme, perforin, and IL-2, indicating their potent cytotoxic responses (**Figures 4E, F**). The crucial role of helper T cell immunity in virus infection control was highlighted in our study. Frequencies of TNF-α, IFN-γ, IL-4, and IL-6 secreted by CD4 T cells exhibited a significant elevation under the influence of the heterologous booster, indicating T cell polyfunctionality. Levels of IL-4 secreted by CD4 T cells were comparable to those induced by homologous boosters (**Figures 4G, H**), underscoring that heterologous vaccination elicits a well-balanced Th1/Th2 vaccine response.

### 3.7 Phenotypical and functional characteristics of rAdMev induced memory T cells

**Figure 5:**
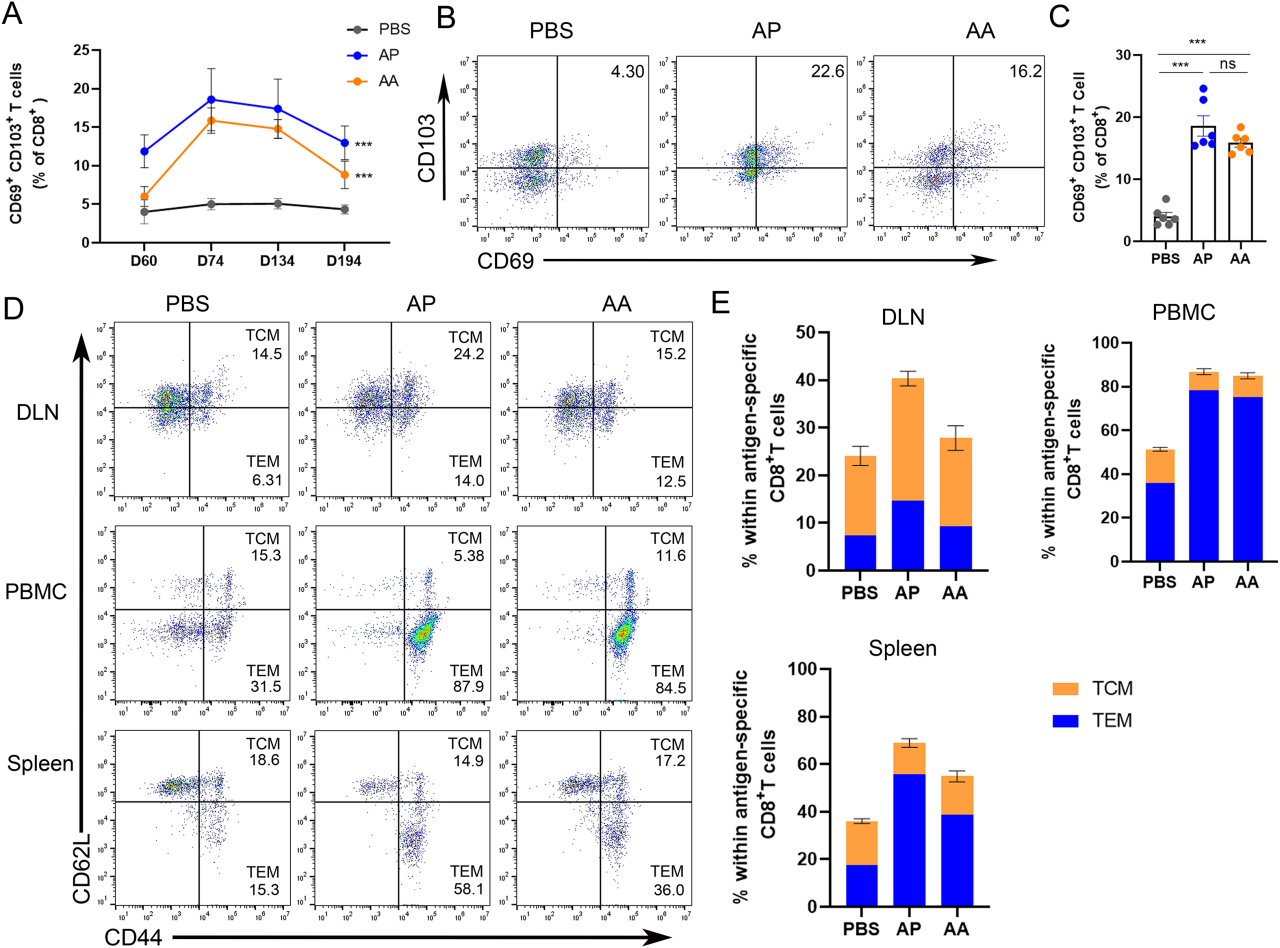
Phenotypic heterogeneity of rAdMev-induced CD8 T cells. **(A)** On days 0, 14, 74 and 134 after the second immune dose, the changing trends of tissue-resident memory T cells in the draining lymph nodes were monitored. **(B, C)** On the 14 days after the booster immunization, the formation of tissue-resident memory T cells in the draining lymph nodes was evaluated. **(D, E)** The lymph nodes, peripheral mononuclear cells, and spleen cells of C57BL/6 mice were collected 14 days after the second injections of either homologous or heterologous booster immunizations. Subsequently, cell-surface molecular staining for CD44 and CD62L on CD8^+^ T cells was performed to evaluate the activation of central memory T cells (TCM) and effector memory T cells (TEM). Each experiment was independently conducted at least three times, n=6. Statistical differences were assessed with One-Way ANOVA followed by Tukey’s test. ^***^P < 0.001.

To assess the phenotypical and functional characteristics of CD8 T cells specifically elicited by rAdMev through homologous or heterologous immunization, mice were immunized with rAdMev and Mev on day 60, respectively (**Figure 5**). The evaluation of specific CD8 T cell responses within the draining lymph nodes was performed via flow cytometry at days 0, 14, 60, and 120 after the administration of the second booster immunization.

Our study revealed that 14 days after the second immunization, a significant number of specific CD8 T cells in the lymph nodes of heterogeneously inoculated mice prominently expressed CD69 and CD103, potentially serving as early indicators of CD8 T lymphocyte residency. Over time, the expression levels of tissue resident memory T cells gradually decreased, eventually returning to baseline levels by day 194 (**Figures 5A–C**). Tissue-resident memory T cells (TRM cells) in peripheral tissue locations can originate from either TEM or TEff cells, guided by tissue-specific factors influencing their migration to these sites (46). It is also plausible that TCM cells may undergo differentiation into TRM cells within lymphoid sites. Hence, we investigated the formation of a diverse subset of T cells within the draining lymph nodes precisely 14 days after the second immunization, based on the migration dynamics from TEM and TCM cells toward TRM. In the memory phase, rAdMev-induced T cell phenotypic traits diverged in different tissues (**Figures 5D, E**). Homologous and heterologous vaccination-induced CD8 T cells within the lymph nodes exhibited fairly mixed phenotype-sharing features of both central memory T cells (CD44^+^, CD62L^+^) and effector memory T cells (CD44^+^, CD62L^-^). As anticipated, specific CD8 T cells induced by heterologous vaccination appeared predominantly as effector memory-like phenotypes in peripheral blood and spleen. Both homologous and heterologous vaccinations demonstrated a comparable ability to induce effector memory T cells (**Figure 5E**).

### 3.8 Multiplexed quantitative analysis of whole proteome during memory T cell activation after homologous or heterologous immunization

**Figure 6:**
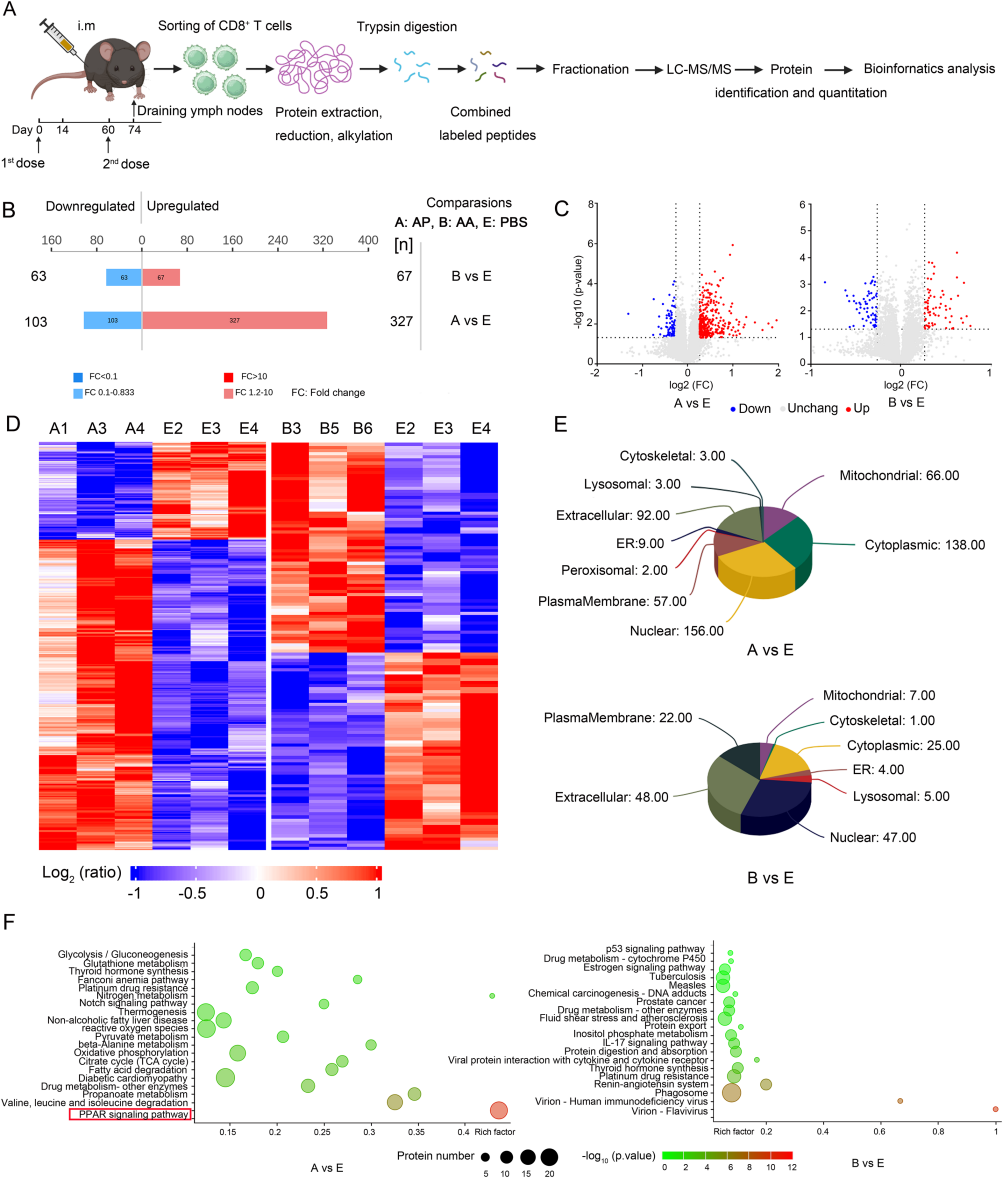
Quantitative proteomics identifies differential induction of proteins during memory T cell activation. **(A)** Experimental scheme. CD8 T cells were purified from the draining lymph nodes of mice 14 days after the second immunization doses, collected and processed by protein extraction and digestion. Each of the three samples had its peptide library labeled with a specific TMT label. These labeled pools were uniformly mixed based on cell numbers and subsequently analyzed using LC-MS/MS for protein quantification. **(B)** The number of up-regulated and down-regulated proteins in the AP group and AA group compared with the PBS group. **(C)** Analysis of substantial protein disparities among the groups. Proteins with significantly up-regulated differential expression are highlighted in red, those with significantly down-regulated expression are represented in blue, while gray dots indicate proteins exhibiting no discernible differential alterations. **(D)** Cluster analysis of differentially expressed proteins. **(E)** Subcellular localization analysis of differentially expressed proteins. **(F)** Pathway categories enriched for proteins differentially expressed by heterologous and homologous vaccination. Fisher’s Exact Test to obtain the significance of the difference between the two comparison groups, to find all the pathway categories of differentially expressed protein enrichment (*P* value < 0.05).

To gain deeper insights into the molecular mechanisms underlying the activation of memory T cells following homologous or heterologous immunization with rAdMev, CD8^+^ T cells were isolated from mouse draining lymph nodes 14 days after second immunizations for quantitative proteome sequencing. We used tandem mass spectrometry coupled with isobaric peptide labeling to quantify alterations in the cellular proteome during memory T cell activation (**Figure 6A**). In the AP group, a total of 430 proteins exhibited differential expression, with 327 proteins being up-regulated and 103 proteins down-regulated, compared to the PBS group. In contrast, the AA group showed differential expression in 130 proteins, comprising 67 up-regulated and 63 down-regulated proteins (**Figure 6B**). To emphasize the significance of protein differences among the three groups (PBS group, AA group, and AP group), we generated a volcano plot utilizing two key factors: fold change in expression (FC) and the P-value (T-test) of proteins in the comparative analysis group. The results, indicated that the heterologous vaccination group exhibited more pronounced significant differences (**Figure 6C**). The reproducibility of these results was further confirmed by cluster analysis based on adjusted P-values less than 0.05 and log2 fold changes greater than 1 in absolute value (**Figure 6D**). To delve deeper into the functional attributes of cellular proteins, subcellular localization analysis of all differentially expressed proteins was conducted employing the subcellular structure prediction software CELLO (42, 47). Differentially expressed proteins induced by heterologous immunization primarily exhibited localization within the nucleus, cytoplasmic, and mitochondrial, whereas those induced by homologous immunization were predominantly localized in the extracellular and cytoplasmic (**Figure 6E**). In order to more intuitively observe the alterations in the differentially expressed proteins induced by heterologous vaccine administration in memory T cells, as well as their involvement in metabolic pathways, we compared all the differentially expressed proteins with the entire proteome of the reference species. The research findings elucidate that differentially expressed proteins in memory T cells are enriched primarily in the PPAR signaling pathway (**Figure 6F**).

### 3.9 Heterologous vaccination elicits memory T cell activation to initiate a synchronized process of mitochondrial biogenesis

**Figure 7:**
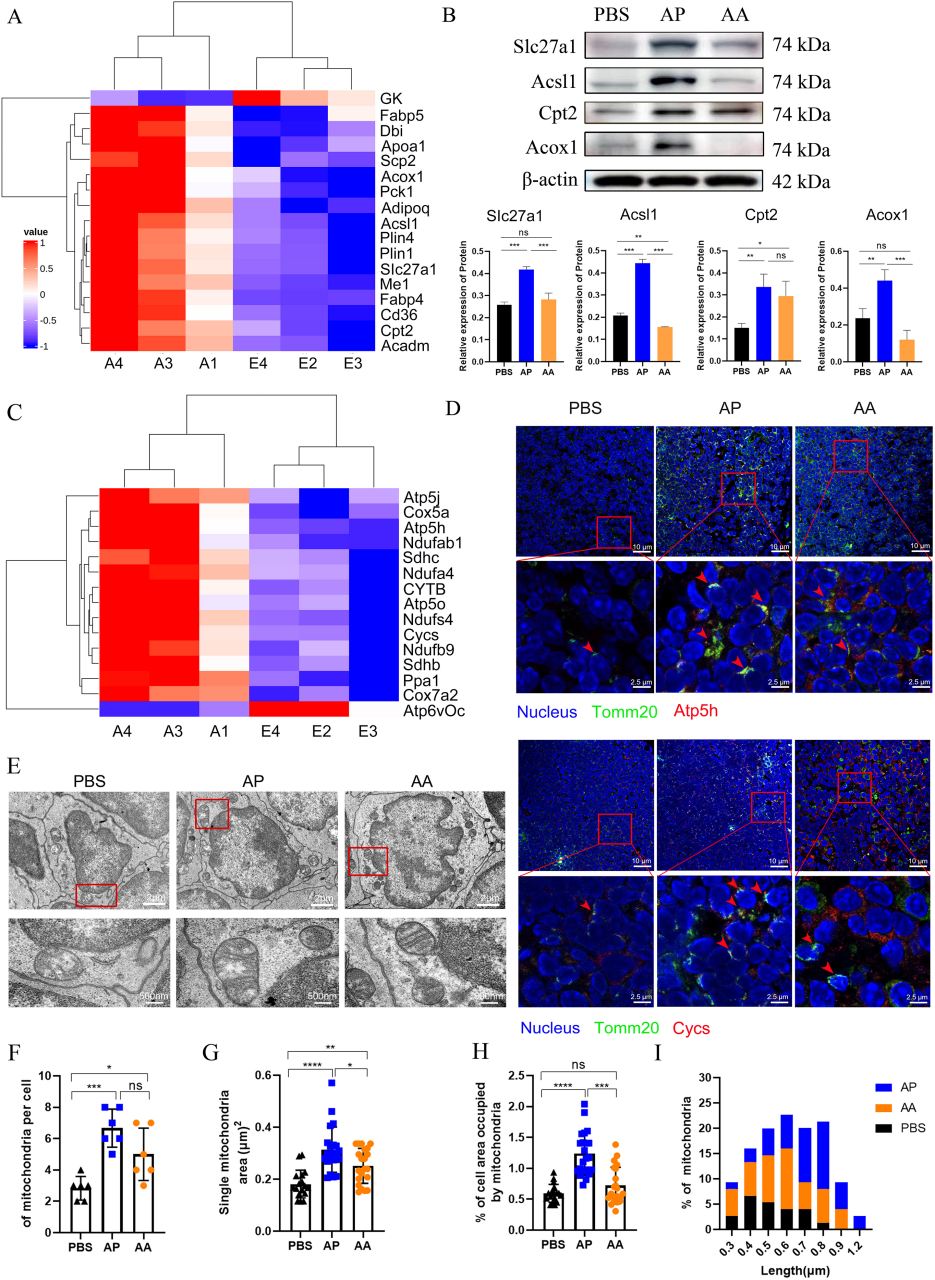
Activation of memory T cell initiates a synchronized program of mitochondrial biogenesis and bioenergetics. **(A)** Heatmap representing the expression of proteins associated with the PPAR pathway. **(B)** Key proteins involved in fatty acid transport and oxidation within the PPAR pathway were quantified by Western blotting, with β-actin utilized as a loading control. **(C)** Heatmap representing the expression of proteins associated with oxidative phosphorylation in mitochondria. **(D)** Immunofluorescence staining was employed to assess mitochondrial activation in the lymph nodes of mice following immunization, with Tomm20 serving as the marker for mitochondrial localization. **(E)** Representative EM micrographs of mitochondria after homologous and heterologous vaccination (n=30 images per group, 6 mice in each group), with a scale bar of 2 μm, and magnification show a scale bar of 200 nm. **(F-H)** Quantification of mitochondrial electron micrographs (6 mice per group) showed the number of mitochondria per cell **(F)**, the area of single mitochondria **(G)**, and the percentage of cell area occupied by mitochondria **(H)**. **(I)** Quantification of mitochondrial length in EM micrographs. All experiments were performed at least three times independently, and the results were expressed as mean ± SEM. Statistical analysis was performed using One-Way ANOVA followed by Tukey’s test. ^*^P < 0.05, ^**^P < 0.01, ^***^P < 0.001, ^****^P < 0.0001.

To comprehensively elucidate the process of mitochondrial biogenesis during memory T cell activation, we utilized proteomics to analyze the energy metabolism of memory T cells in draining lymph nodes following heterologous vaccination. Fatty acid β-oxidation plays a pivotal role in mitochondrial activation, that fuels the respiratory chain (RC) complexes of the tricarboxylic acid cycle (TCA) and OXPHOS, was a focal point of our investigation (48). As anticipated, heterologous vaccination activated the PPAR signaling pathway upregulating the expression of proteins involved in fatty acid degradation, transport, and oxidation compared to the PBS group (**Figure 7A**). In contrast, homologous vaccination demonstrated a weaker activation effect (**Figure 7B**). The proteins associated with mitochondrial oxidative phosphorylation, including ATP5h and Cycs, were significantly increased, in the heterologous vaccination group compared to the PBS group, while the effect of homologous immunization was relatively modest (**Figures 7C, D**).

To quantify these remarkable variations, we examined mitochondria by Electron Microscopy (EM) (**Figure 7E**). As anticipated, both heterologous and homologous vaccination-induced mitochondrial replication within cells, resulting in a 4-fold and 2-fold increase in the number of mitochondria (**Figure 7F**), along with a 3-fold and 1.5-fold expansion in the mitochondrial area (**Figure 7G**). The relative area occupied by mitochondria in the cytoplasm was significantly increased in the heterologous vaccination group (**Figure 7H**).

Different immunization methods led to varying morphological alterations in the mitochondria. Cells in the PBS group contained fragmented and round mitochondria, with the length predominantly concentrated at 0.4 μm. In the heterologous vaccination group, the mitochondrial ultrastructure also changed significantly, featuring invaginated inner membranes with tight cristae, elongated and fused structures. This elongated intermediate was about 0.8 μm, possibly associated with higher supramolecular organization of respiratory chain complexes in supercomplexes (49). In the homologous vaccination group, the mitochondrial length was mainly concentrated at 0.6 μm (**Figure 7I**). These studies reveal that heterologous vaccination induces robust and synchronized mitochondrial biogenesis during the activation of memory T cells. Importantly, heterologous vaccination proves significantly more effective than homologous vaccination in this context (**Figure 7I**).

### 3.10 Heterologous vaccination induces robust energy metabolism in mitochondria

In the process of metabolic reconfiguration in T cells, mitochondria are far from inert. T cell activation, for instance, stimulates metabolic flux within the TCA cycle, resulting in citrate production for lipid biosynthesis and providing electron donors for the electron transport chain (ETC) (49). Activation of the ETC generates energy and plays a crucial role in signaling events during T-cell activation (50).

**Figure 8:**
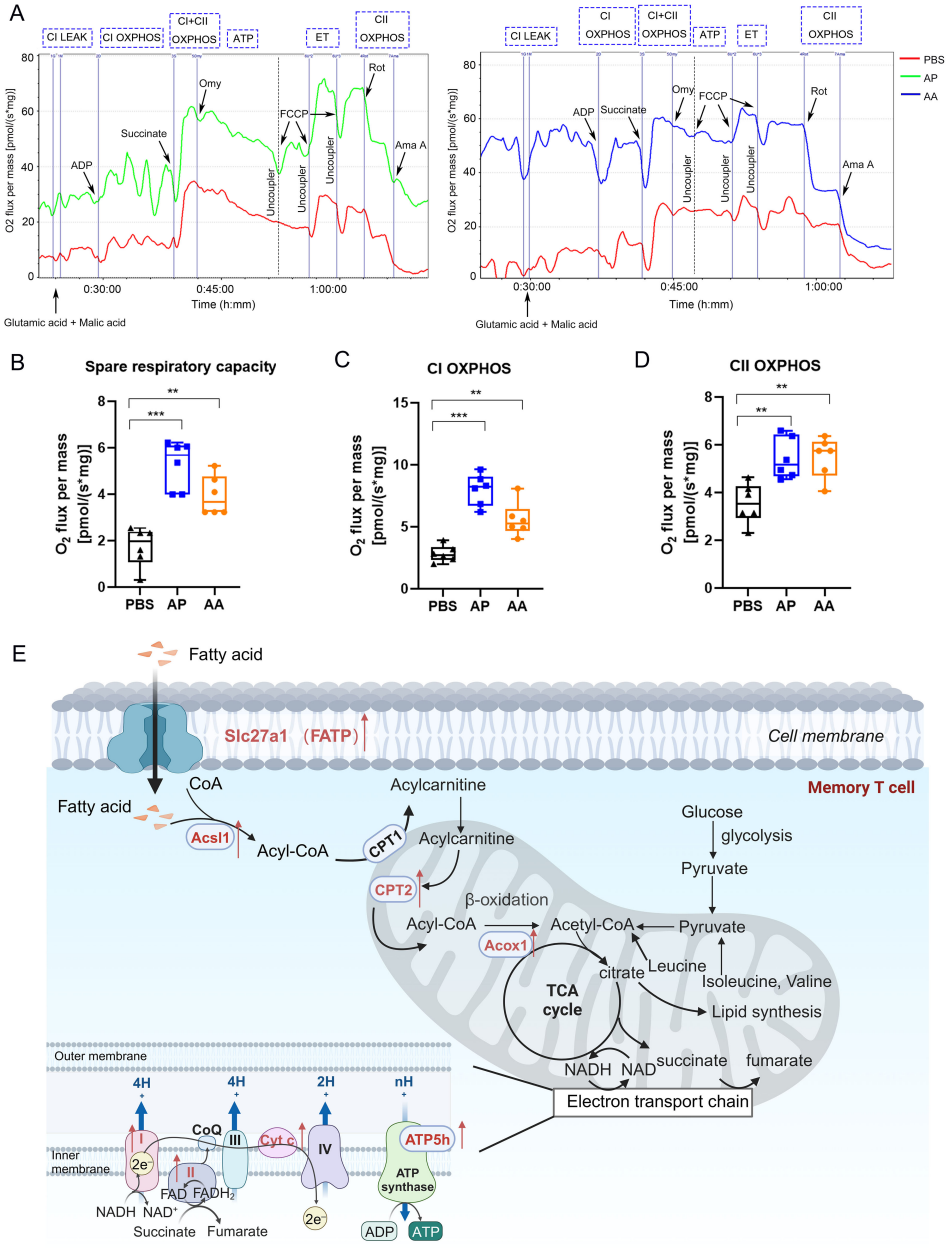
Mitochondrial energy metabolism in memory T cells. **(A)** The oxygen consumption rate of T cells after vaccination. **(B)** Spare respiratory capacity was calculated by analyzing changes in the oxygen consumption rate. Oxidative phosphorylation capacity of Complex I **(C)** and Complex II **(D)** in the mitochondrial electron transport chain. **(E)** Schematic illustrating of mitochondrial energy metabolism. Each group consisted of 6 mice, and all results were presented as mean ± SEM. At least three independent experiments were conducted. Statistical analysis was performed using One-Way ANOVA followed by Tukey’s test. Significance levels are denoted as follows ^**^P < 0.01, ^***^P < 0.001.

To assess mitochondrial electron transport induced by heterologous vaccination, we isolated mitochondria from lymph nodes 14 days after second immunizations and conducted measurements using the O2K cell energy metabolism analysis system (**Figures 8A, E**). The results showed that mitochondrial activation was induced by heterologous immunization, and the basal respiration of the cells reached its maximum capacity, which was three times higher than that of resting cells in the PBS group (**Figure 8B**). Subsequently, we measured the oxidative phosphorylation capacity of mitochondrial Complex I (CI) and Complex II (CII). The findings indicated that compared to the PBS group, both heterologous and homologous immunizations enhanced the oxidative phosphorylation capacity of CI and CII. Notably, the activation effect of heterologous immunization was significantly more pronounced than that of homologous immunization (**Figures 8C, D**).

## 4 Discussion

CMV reactivation poses a major challenge to hematopoietic cell transplantation by causing severe complications, impairing immune reconstitution, and increasing morbidity in immunocompromised recipients, diminishing the full curative potential of this successful cancer therapy. Indeed, the pentameric complex (gH/gL/UL128/UL130/UL131A) targeted by Moderna and MSD’s mRNA-based HCMV vaccines is a promising candidate for eliciting potent neutralizing antibodies, particularly against epithelial and endothelial cell infection (51). However, HCMV-specific antibodies often exhibit low affinity and limited neutralizing activity, allowing herpesviruses to evade host immune defenses (52). Immunotherapy based on infusion of limited numbers of CMV-specific T cells restored durable, functional antiviral immunity in HCT recipients, thus bridging the critical post-transplant period of high susceptibility to uncontrolled viremia (53). T-cell immunity targeting HCMV is effective in suppressing latent viral reinfection, the immunodominant epitopes induced by natural infection may be suboptimal (54). Thus, an immunoinformatics approach simplifies the development of vaccines based on dominant T cell epitopes. In this study, the rAdMev designed using immunoinformatics incorporates CD8^+^, CD4^+^ T cell, and B cell epitopes, collectively eliciting a robust antiviral immune response in the body. Fascinatingly, we observed that heterologous vaccination with rAdMev could mediate the metabolic reprogramming of memory T cells, concurrently enhancing mitochondrial biogenesis, thereby effectively preventing CMV infection.

Jyotirmayee Dey et al. highlighted a major hurdle in vaccine development the risk of induced hypersensitivity (55). This condition occurs when vaccination triggers an allergic reaction in the body instead of the desired immune response against the virus (56). Our study has discovered that all candidate peptides exhibit a good safety profile (**Supplementary Figure S2**). For MHC class I binding peptides, the coverage rate in the European population is as high as 96.34%. Similarly, for class I and class II binding peptides, the top three geographical areas for the immune responses were Europe, North America, and Oceania, with coverage rates of 98.52%, 95.63%, and 93.21%, respectively. The population coverage of MHCII class combined peptides stands at 59.52% in Europe, which is lower than MHCI and Class combined. Therefore, our findings suggest that MHCI and class-combined binding peptides may be effective vaccine targets for diverse populations worldwide.

Toll-like receptors belong to the pattern recognition receptor family, and they are expressed on all innate immune cells (including dendritic cells and macrophages), and most non-hematopoietic cells (57). They play a crucial role in recognizing pathogens and initiating innate immune responses (58, 59). Previous studies have demonstrated that the stimulation of TLRs by structural proteins from various viruses activates signal transduction pathways, leading to the production of various inflammatory cytokines in response to viral infection (60–62). Immunodominant epitope-peptides could activate CD8 T cells and NK cells simultaneously via interacting with TLR4 receptors on the surface of DCs to suppress tumors *in vivo* (63). Bioactive peptide can also directly bound to TLR4 in the extracellular region to facilitate the inflammatory factor secretion (64). Therefore, interactions between toll-like receptors and antigen molecules are pivotal in the initial activation of the immune system. We confirmed the stable interaction between the candidate peptides and TLR4 through molecular docking and molecular dynamics simulations. This sheds light on the crucial roles of electrostatic and van der Waals energies in the binding process. Research has unveiled that TLR4 exhibits the lowest binding energy (-325.61 kcal/mol) and the most stable contact capability with synthetic polypeptides containing multiple antigenic epitopes. We conducted further validation of the activation of innate immune cells (DC2.4 and RAW264.7) by the candidate peptides *in vitro* and observed that Mev has the capacity to bind firmly to immune receptors, leading to a substantial induction of innate immune cell activation compared to a single peptide. These findings align with the computer simulations results.

Recombinant adenovirus vectors encoding pathogen proteins serve as a secure and efficient immunogenic vaccine delivery platforms, capable of eliciting robust and specific cellular and humoral immune responses (65). Consistent with the findings by Li et al. (66), our observations revealed the activation of migrating DCs, resident DCs, and macrophages in the draining lymph nodes at least 7 days after immunizing mice with the rAdMev vaccine. This induction was accompanied by a significant release of inflammatory factors and chemokines. Adenovirus vectors have been recognized as potent triggers of T-cell proliferation (67). However, repeated homologous vaccination, impedes the augmentation of the cellular immune response due to anti-vector immunity (68).

Spencer et al. discovered that employing a heterologous prime-boost vaccination strategy, combining diverse antigen delivery systems enhances the immune response (69). In line with prior studies, we used rAdMev and Mev vaccines for homologous and heterologous boosters on day 60, respectively. In contrast to homologous vaccination, heterologous vaccination induces a substantial expansion of antigen-specific T cells at 14 days after booster immunization, marked by high expression of CD107a and CD69 on the surface of CD8 T cells. This stimulation results in multifunctional CD8 T cell responses characterized by the secretion of TNF-α, IFN-γ, IL-2, Granzyme B, and perforin. Additionally, it leads to the secretion of IFN-γ, TNF-α, IL-6, and IL-4 in CD4 T cells, along with the secretion of Th1-type HCMV-specific IgG2a antibodies. One limitation of our immunogenicity analysis is that T-cell responses were assessed using a pooled peptide approach. Therefore, quantifying the frequency of antigen-specific T cells by ELISpot and clarifying the immune dominance levels among the selected antigens remain important goals for future research.

The above data highlight that heterologous vaccination with the rAdMev vaccine can elicit a robust cellular immune response. Adenovirus vaccines not only elicit multifunctional antibodies capable of mediating viral neutralization but also drive other antibody-dependent effector functions (70). Heterologous vaccination with the rAdMev vaccine produced high levels of specific IgG following the second booster dose. Furthermore, it rapidly generated high-affinity specific IgG and neutralizing antibodies upon antigen re-stimulation on the 60th day, with the IgG titer significantly surpassing that of the homologous vaccination group.

The establishment of durable, long-term immune protection is a key goal in vaccination strategies, with immune memory being a major feature of the adaptive immune response. The success of these strategies hinges on the quantity and quality of the memory T cell population (71), making it imperative for vaccines to elicit a robust population of effector memory T cells.

Fourteen days after the second immunization, the heterologous booster of the rAdMev vaccine resulted in a substantial increase in high-affinity effector memory T cells (CD44^hi^, CD62L^lo^) in peripheral blood mononuclear cells and the spleen. This increase led to significant memory inflation, highlighting the effectiveness of the vaccination approach. Notably, memory T cells induced by the rAdMev vaccine exhibited a mixed central and effector-memory T cell phenotype (CD62L^+/-hi^, CD44^hi^) in draining lymph nodes, consistent with findings from previous studies (12).

Tissue-resident memory cells act as sentinels within tissues, orchestrating a cyclic memory T-cell response upon antigen encounter and enhancing local antiviral immunity (72). Following the rAdMev vaccine booster, a substantial number of tissue-resident memory T cells were observed in the lymph nodes. These cells persisted from the 14th day after boost immunization and remained detectable for up to 120 days thereafter. Collectively, these distinct subsets of memory T cells elicited by the rAdMev vaccine work in concert to establish a multifaceted defense strategy, contributing to effective immunity during HCMV reinfection.

Previous studies have shown that memory CD8 T cells mediate long-term immune protection, attributed to their heightened proliferation capacity, self-renewal potential, and unique metabolic adaptations that maintain cell viability (18). Notably, these memory CD8 T cells rely on fatty acid oxidation and oxidative phosphorylation to fulfill their metabolic requirements. In alignment with these findings, our proteomic analysis of memory T cells induced by heterologous vaccination with the rAdMev vaccine revealed significant enhancements. The vaccine effectively increased mitochondrial mass and elicited a higher SRC, a critical feature in natural CD8 T cell memory development. As observed in our study, augmented mitochondrial mass provides a survival advantage (17). Moreover, the heterologous vaccination with the rAdMev led to increased oxygen consumption in complex I and complex II of the respiratory chain. This enhancement indicates an elevated capacity for mitochondrial oxidative phosphorylation, meeting the metabolic demands required for enhanced memory T cell proliferation. Nonetheless, vaccination relies on human HLA. Nevertheless, our experiments encountered several limitations. The high specificity of HCMV infection poses a significant challenge as there is no suitable animal model to conduct challenging experiments. In our future research endeavors, we would evaluate the immunogenicity of the recombinant adenovirus vaccine using human cell models.

Collectively, we have developed a recombinant adenovirus vaccine utilizing multiple epitopes of human cytomegalovirus. This vaccine exhibits the capability to induce the maturation of APCs *in vitro* and *in vivo*. It facilitates dendritic cell migration and macrophage polarization, fostering a synergistic interaction between cellular and humoral immunity. Furthermore, heterologous vaccination with the rAdMev demonstrates a noteworthy role in triggering mitochondrial proteome remodeling and biogenesis during memory T cell activation. Our findings underscore the potential of the rAdMev vaccine as an effective and safe immunization strategy against HCMV.

## Data Availability

The datasets used and/or analysed during the current study are available from the corresponding author on reasonable request. Proteomic analysis raw data have been deposited in the Mendeley Data (DOI:10.17632/9kxptxbgpb.1).

## Supplementary material

Supplementary Table 1 Prediction of potential HCMV cytotoxic T lymphocyte epitopes.

Supplementary Table 2 Prediction of potential HCMV helper cytotoxic T lymphocyte epitopes.

Supplementary Table 3 Prediction of potential HCMV line B-cell epitopes.

Supplementary Table 4 Prediction of the dominant epitope sequences for mice.

Supplementary Figure 1 Analysis of HLA Coverage in Population. **A** and **B** Predicted coverage of the dominant Epitope peptides in 16 regions of the world. **C** Average number of epitope hits/HLA combinations recognized by the population. **D** Minimum number of epitope hits/HLA combinations identified by 90% of the population.

Supplementary Figure 2 Safety Assessment of rAdMev Vaccine. **A** An Elisa was used to detect changes in ALT, AST, UA, CK, and LDH in mouse serum after boost immunition. Mouse body weight (**B**) and food intake (**C**) were monitored for one week following the second immunization. There were 6 mice in each group.

Supplementary Figure 3 Gating strategy. **A** Innate immune cell subtype gating strategies. **B** The gating strategy was used to identify CD3^+^ T cells, CD8^+^ T cells, CD4^+^ T cells, and their secreted cytokines. **C** Gating Strategy for the Identification of CD3^+^ T cells, CD8^+^ T cells, CD44^+^ CD62L^-^ (TEM) CD8 T cells, CD44^+^ CD62L^+^ (TCM) CD8 T cells, and CD103^+^ CD69^+^ (TRM) CD8 T cells.

Supplementary Figure 4 Main KEGG pathways enriched by differential proteins between heterologous vaccination, homologous vaccination, and PBS group. A Top 12 up and down-regulated KEGG pathways induced by heterologous and homologous vaccination. Fisher’s Exact Test to obtain the significance of the difference between the two comparison groups, to find all the pathway categories of differentially expressed protein enrichment (*P* value < 0.05).
