# Supplementary figures and images for: Optimized multi-epitope neoantigen human cytomegalovirus vaccine based on adenovirus vectors elicits potent antiviral immunity
Source: Front Immunol. 2025 Nov 21;16:1658220. doi: 10.3389/fimmu.2025.1658220 (PMC12678279; doi:10.3389/fimmu.2025.1658220)

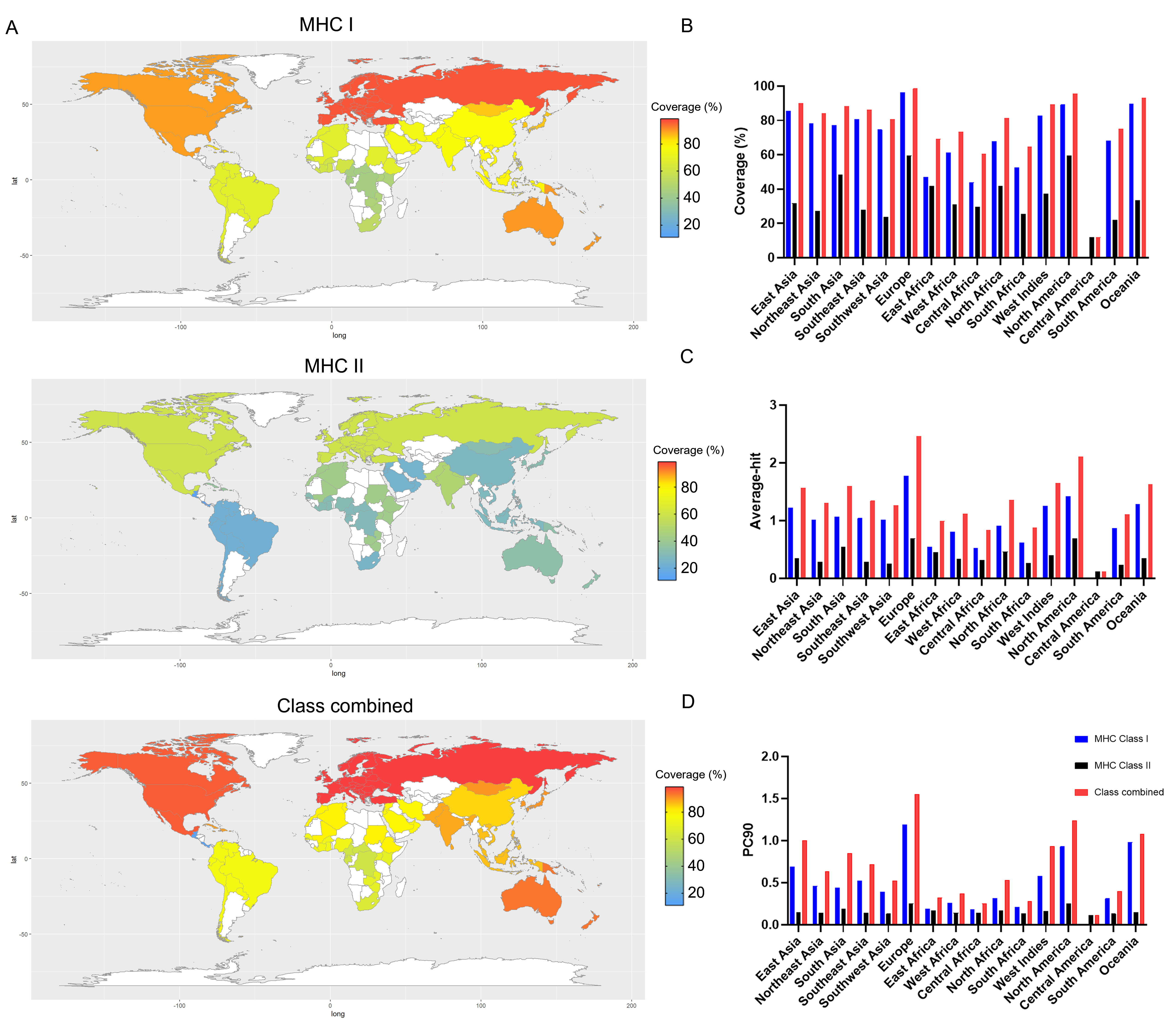

Supplement: Supplementary Figure 1 — Analysis of HLA Coverage in Population. A and B Predicted coverage of the dominant Epitope peptides in 16 regions of the world. C Average number of epitope hits/HLA combinations recognized by the population. D Minimum number of epitope hits/HLA combinations identified by 90% of the population. [file Image1.tif]

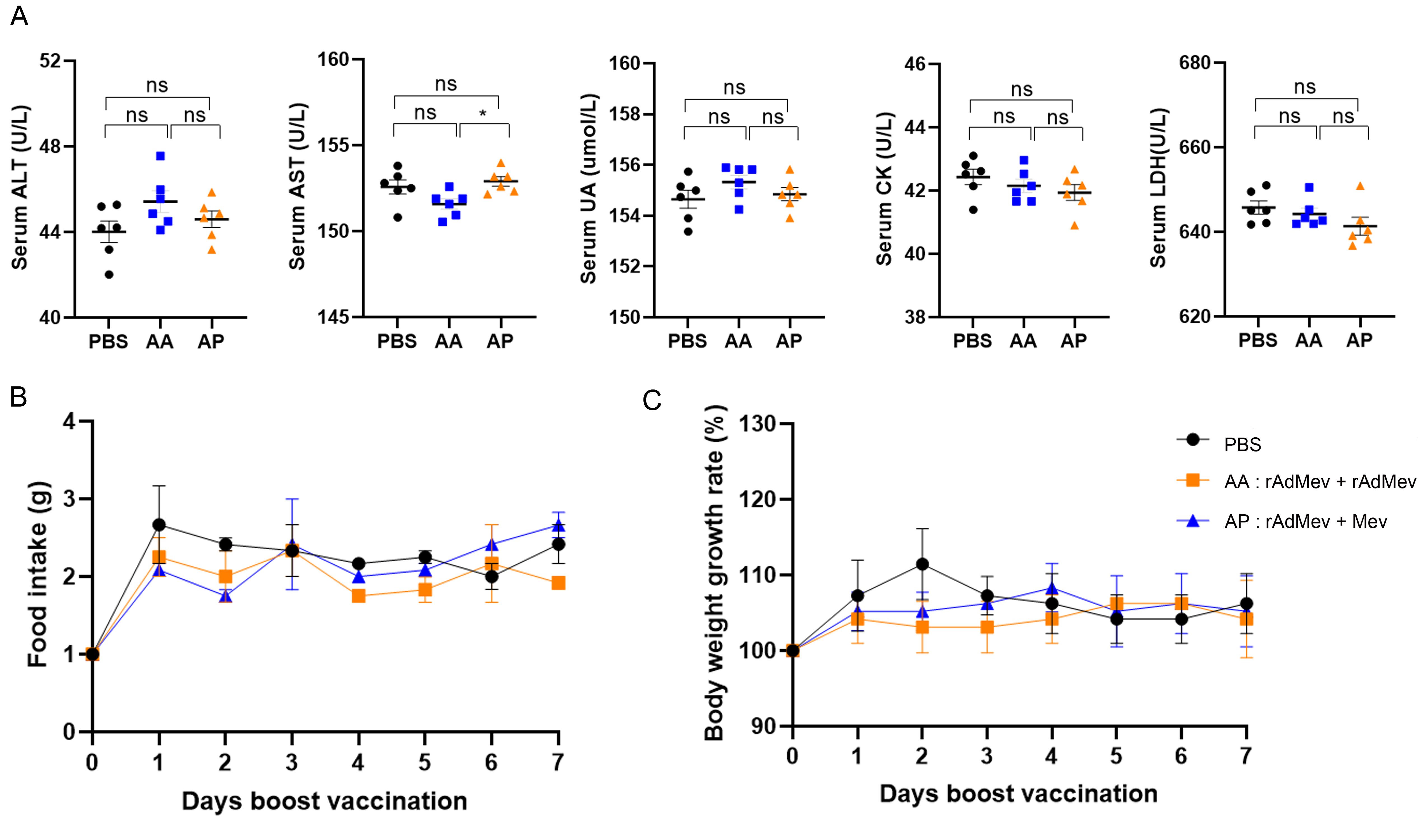

Supplement: Supplementary Figure 2 — Safety Assessment of rAdMev Vaccine. A An Elisa was used to detect changes in ALT, AST, UA, CK, and LDH in mouse serum after boost immunition. Mouse body weight (B) and food intake (C) were monitored for one week following the second immunization. There were 6 mice in each group. [file Image2.tif]

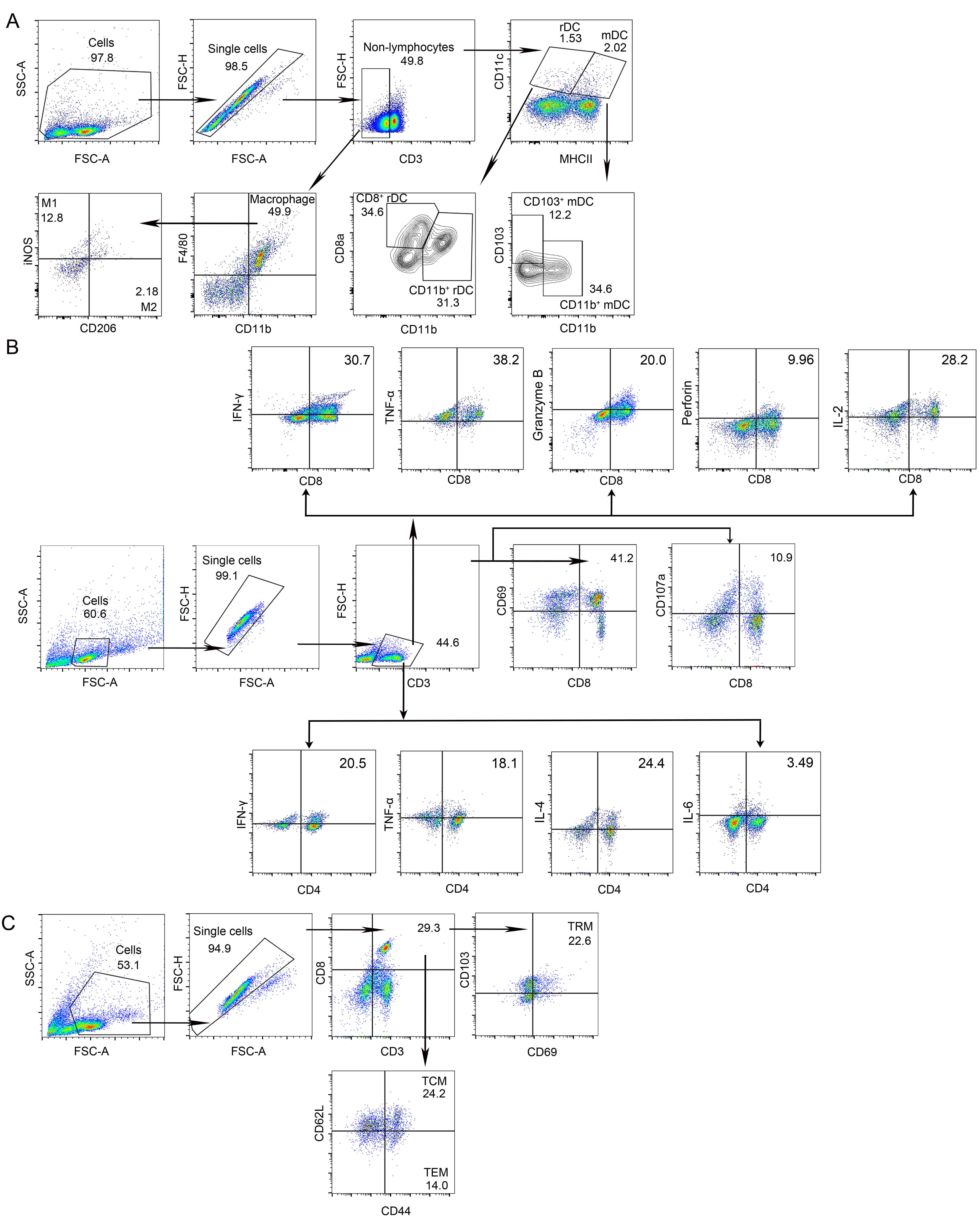

Supplement: Supplementary Figure 3 — Gating strategy. A Innate immune cell subtype gating strategies. B The gating strategy was used to identify CD3+ T cells, CD8+ T cells, CD4+ T cells, and their secreted cytokines. C Gating Strategy for the Identification of CD3+ T cells, CD8+ T cells, CD44+ CD62L- (TEM) CD8 T cells, CD44+ CD62L+ (TCM) CD8 T cells, and CD103+ CD69+ (TRM) CD8 T cells. [file Image3.tif]

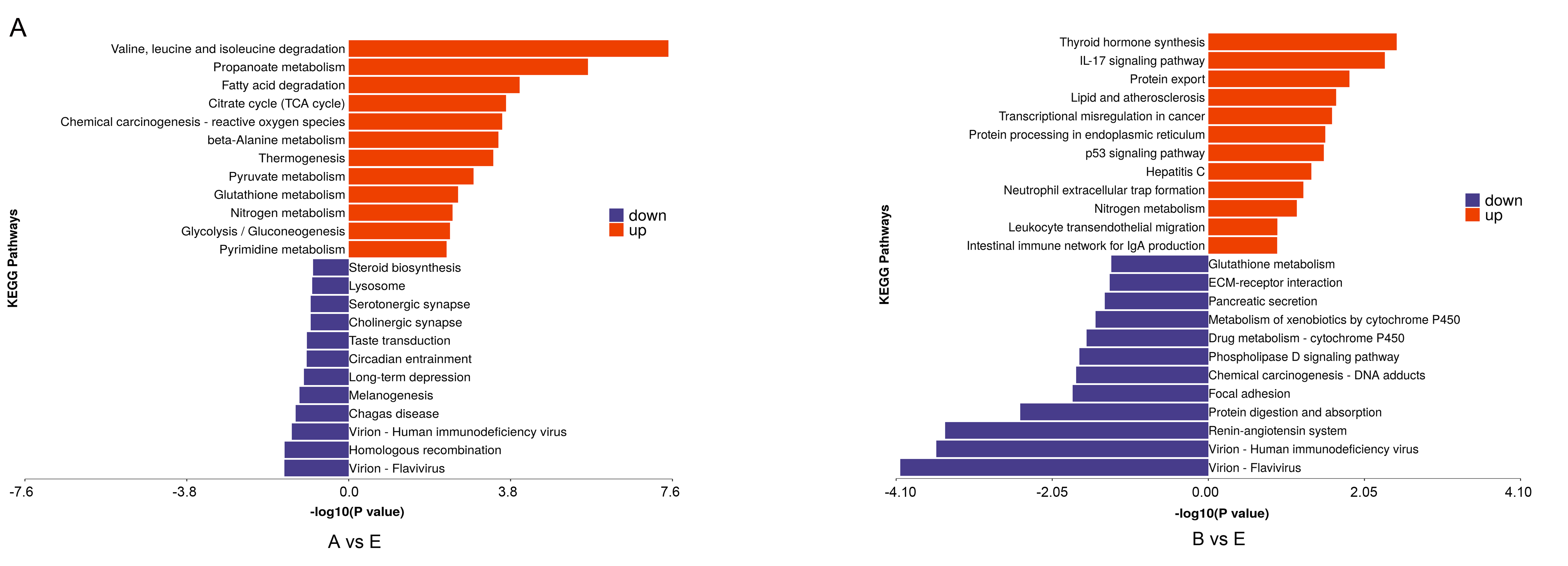

Supplement: Supplementary Figure 4 — Main KEGG pathways enriched by differential proteins between heterologous vaccination, homologous vaccination, and PBS group. A Top 12 up and down-regulated KEGG pathways induced by heterologous and homologous vaccination. Fisher’s Exact Test to obtain the significance of the difference between the two comparison groups, to find all the pathway categories of differentially expressed protein enrichment (P value < 0.05). [file Image4.tif]
